# Tailoring Atomic Ordering Uniformity Enables Selectively Leached Nanoporous Pd‐Ni‐P Metallic Glass for Enhanced Glucose Sensing

**DOI:** 10.1002/advs.202408816

**Published:** 2024-11-05

**Authors:** Yu Lou, Jian Li, Zhongzheng Yao, Zhenduo Wu, Huiqiang Ying, Lan Tan, Sinan Liu, Jianrong Zeng, Ruohan Yu, Hong Liu, Xun‐Li Wang, He Zhu, Si Lan

**Affiliations:** ^1^ Herbert Gleiter Institute of Nanoscience, School of Materials Science and Engineering Nanjing University of Science and Technology Nanjing 210094 China; ^2^ School of Bioscience and Technology Chengdu Medical College Chengdu 610500 China; ^3^ City University of Hong Kong (Dongguan) Dongguan 523000 China; ^4^ Department of Physics City University of Hong Kong 83 Tat Chee Avenue, Kowloon Hong Kong SAR China; ^5^ Shanghai Synchrotron Radiation Facility, Shanghai Advanced Research Institute Chinese Academy of Sciences Shanghai 201204 P. R. China; ^6^ Shanghai Institute of Applied Physics Chinese Academy of Sciences Shanghai 201800 P. R. China; ^7^ State Key Laboratory of Advanced Technology for Materials Synthesis and Processing Wuhan University of Technology Wuhan 430070 P. R. China; ^8^ State Key Laboratory of Bioelectronics School of Biological Science and Medical Engineering Southeast University Nanjing 210096 China

**Keywords:** electrochemical glucose sensor, medium‐range order, metallic glass, nanostructured

## Abstract

Constructing nanostructures, such as nanopores, within metallic glasses (MGs) holds great promise for further unlocking their electrochemical capabilities. However, the MGs typically exhibit intrinsic atomic‐scale isotropy, posing a significant challenge in directly fabricating anisotropic nanostructures using conventional chemical synthesis. Herein a selective leaching approach, which focuses on tailoring the uniformity of atomic ordering, is introduced to achieve pore‐engineered Pd‐Ni‐P MG. This innovative approach significantly boosts the number of exposed active sites, thereby enhancing the electrochemical sensitivity for glucose detection. Electrochemical tests reveal that the nanoporous Pd‐Ni‐P MG exhibits high sensitivity (3.19 mA mm⁻¹ cm⁻^2^) and remarkable stability (97.7% current retention after 1000 cycles). During electrochemical cycling, synchrotron X‐ray pair distribution function and X‐ray absorption fine structure analyses reveal that the distance between active sites decreases, enhancing electron transport efficiency, while the medium‐range ordered structure of the Pd‐Ni‐P MG remains stable, contributing to its exceptional glucose sensing capabilities. A microglucose sensor is successfully developed by integrating the nanoporous Pd‐Ni‐P MG with a screen‐printed electrode, demonstrating the practical applicability. This study not only offers a new avenue for the design of highly active nanoporous MGs but also sheds light on the mechanisms behind the high electrochemistry performance of MGs.

## Graphical abstract

A “bottom‐up” atomic‐level engineering is proposed for the first time, based on the dynamic evolution during the crystallization of metallic glasses (MGs). By tailoring atomic ordering uniformity, the nanoporous MGs are constructed, which have significantly improved electrochemical sensing performance. Additionally, the study is elucidated the correlation between electrochemical ability and the atomic short‐to‐medium range order structures.

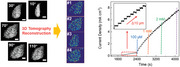

## 1 Introduction

Diabetes, recognized as one of the top ten chronic diseases globally, presents considerable health threats to individuals.^[^ ^1^, ^2^, ^3^, ^4^ ^]^ At the core of all diabetes therapies is the control of glucose levels, which requires high‐sensitive monitoring of glucose concentration. Currently, the majority of glucose sensors rely on enzymatic reactions, known for their high selectivity and rapid response rates.^[^ ^5^, ^6^ ^]^ However, the inherent limitations of enzymes, such as high cost and poor reproducibility, pose significant challenges.^[^ ^6^, ^7^, ^8^, ^9^ ^]^ In contrast, nonenzymatic electrochemical glucose sensors have gained increasing attention due to their high sensitivity, low detection limits, and excellent reversibility.^[^ ^10^, ^11^, ^12^, ^13^ ^]^ However, these sensors often require electrodes made of high‐price noble metals, such as Au, Pt, and Pd as probe materials.^[^ ^14^, ^15^, ^16^ ^]^ Therefore, there is an urgent need to develop electrochemical probe materials with reduced dependence on noble metals while maintaining high reactivity.

Metallic glasses (MGs), also known as amorphous alloys, are characterized by their long‐range atomic disorder,^[^ ^17^, ^18^, ^19^ ^]^ which enables them to have a higher Gibbs free energy with a greater density of low‐coordinated sites compared to their crystalline counterparts.^[^ ^20^, ^21^, ^22^ ^]^ This unique structure also leads to a higher density of electronic states, facilitating enhanced electron transport and enabling shallow *d*‐band electrons to actively participate in electrochemical reactions.^[^ ^23^ ^]^ Therefore, amorphous alloys hold significant promise in the field of electrochemistry.^[^ ^24^, ^25^, ^26^, ^27^, ^28^ ^]^ However, traditional preparation methods, such as copper mold casting and melt spinning, produce bulk, or ribbon MGs with limited surface area and few exposed active sites, restricting their interaction with reactants.^[^ ^22^, ^29^ ^]^ The construction of nanostructures like nanopore within bulk MG matrix is one way to address this limitation, wherein dealloying is a prevailing method.^[^ ^30^, ^31^ ^]^ However, this method involves the removal of more active elements based on their chemical reactivity, leading to significant compositional changes and, thereby, new challenges, such as crystallization, during the dealloying of MGs. Unlike crystalline alloys, where anisotropic atomic packing leads to dispersion of energy and chemical reactivity,^[^ ^20^, ^32^, ^33^ ^]^ the MGs are statistically isotropic, challenging the direct fabrication of anisotropic nanostructures in the MG matrix using chemical methods. Nonetheless, recent studies have revealed that MGs are not completely isotropic but comprise locally interconnected atomic clusters within short‐ to medium‐range orders.^[^ ^34^, ^35^, ^36^, ^37^ ^]^ For example, our team found that Pd‐Ni‐P MG contains six‐membered tricapped trigonal prism (6M‐TTP)^[^ ^38^ ^]^ clusters formed by tricapped trigonal prisms (TTPs)^[^ ^38^, ^39^ ^]^ rearranging at the proper annealing temperature. This atomic‐scale inhomogeneity has motivated us to construct nanostructures, such as nanopores, by precisely tailoring the atomic ordering.

In this work, we proposed a selective leaching method to fabricate interconnected nanoporous structures in Pd‐Ni‐P MG for enhanced electrochemical performance. By employing appropriate heat treatment, the Pd‐Ni‐P MG precipitates high‐density and more ordered rhombohedral atomic clusters. These clusters are then selectively removed by exploiting the difference in chemical stability between the clusters and the matrix, eventually forming a nanoporous MG structure. Electrochemical tests show that the resulting nanoporous MG exhibits high electrochemical glucose sensing performance, attributed to the optimization of electron transport during electrochemical processes and the stable medium‐range ordered structure. Additionally, we integrated the MG electrode with screen‐printed electrodes to develop a prototype of a nonenzymatic glucose sensor. This study not only introduces a novel approach for the preparation of nanoporous MGs but also offers new insights into the relationship between local structural ordering and the electrochemical reactivity of MGs.

## 2 Results and Discussion

**Figure 1:**
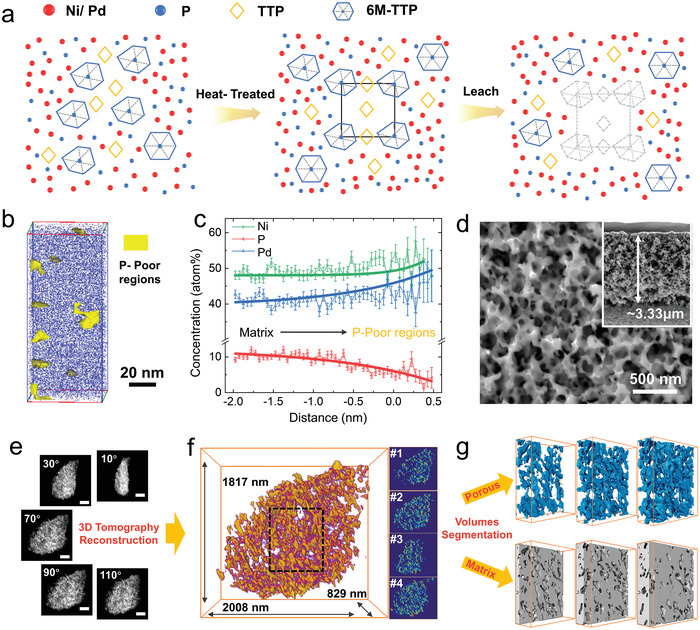
a) Schematic illustration of selective leaching approach. b) ATP‐3D reconstructed P atom maps of the heat‐treated Pd‐Ni‐P. c) 1D concentration profiles through the center of the selected P‐poor region. d) The surface morphology image of nanoporous Pd‐Ni‐P MG by SEM. Inset shows the cross‐section SEM image. e) Representative STEM‐HAADF images at different rotation angles. Scale bar, 50 nm f) Reconstructed nanoporous Pd‐Ni‐P MG and the representative orthoslices (*xy* planes, perpendicular to the *z*‐axis at 300 nm (#1), 360 nm (#2), 420 nm (#3) and 480 nm (#4)). g) Volumes from segmentation, by contrast, corresponding to porous (blue) and matrix (gray).

First, we elucidate the underlying design principles of the selective leaching approach, highlighting how it modulates the atomic ordering to facilitate the formation of porous MGs (see the schematic diagram in **Figure** **1**a). The bulk Pd‐Ni‐P (i.e., Pd_41.25_Ni_41.25_P_17.5_), which serves as the starting material for the pore engineering, contains 6M‐TTP clusters characterized by six interconnecting TTP branches within the medium‐range order (Figure S1, Supporting Information). Additionally, individual TTP clusters are also distributed within the short‐range scale. When subjected to a heating‐quenching process at an appropriate heating temperature (see Figure S2 and Note S2, Supporting Information), the atomic ordering of the Pd‐Ni‐P MG is tailored, leading to the formation of crystalline clusters composed of TTP and 6M‐TTP arranged in a rhombohedral *R*3 symmetric manner (Figure S3, Supporting Information). The next step involves subjecting these heat‐treated samples to a leaching process in a corrosive solution, which selectively removes the more ordered rhombohedral clusters due to their relatively poor corrosion resistance, eventually forming nanopores dispersed within the amorphous alloy matrix.

Figure 1b depicts a 3D reconstruction of the Pd‐Ni‐P MG after the heating‐quenching treatment (heat‐treated Pd‐Ni‐P) using atom probe tomography (APT). The results validate that this process induces compositional segregation, resulting in the formation of P‐poor nanoclusters with sizes ranging from 7 to 25 nm, as illustrated by the yellow regions in Figure 1b. The line‐profile analysis (Figure 1c) reveals a notable shift in the average composition when scanning from the matrix to the nano region, where Pd and Ni enrich while P inversely diminishes. Based on the APT analysis, the average composition of the nanoregions is Pd_44.08_Ni_50.84_P_5.08_, in contrast to the overall average composition of the MG, i.e., Pd_41.39_Ni_48.73_P_9.88_. Note that the content of P obtained from APT is relatively lower than the actual stoichiometry, which arises from the evaporation process during the APT,^[^ ^40^ ^]^ resulting in P loss. As mentioned, the rhombohedral‐type nanocrystals consist of TTP and 6M‐TTP with a composition of (Pd, Ni)_35_:P_6_, whose P content is lower than the target (Pd_41.25_Ni_41.25_P_17.5_). Therefore, the observed P‐poor nanoregions can be assigned to these rhombohedral nanoclusters.

The formation of the nanocrystals has been further validated by a transmission electron microscope (TEM). The initial bulk MG, as observed in the high‐resolution TEM (HRTEM) image (Figure S4a, Supporting Information), does not display any lattice fringe, with the corresponding selected area electron diffraction (SAED) confirming the amorphous nature (Figure S4a inset, Supporting Information). After the heating‐quenching treatment, the nanocrystals with cubic morphology emerge within the amorphous matrix, as evidenced by the SAED image showing bright Bragg rings accompanied by an amorphous halo in the middle (Figure S4b, Supporting Information). From the HRTEM image, the interplanar spacing of ≈13 Å could be observed, corresponding to the (1 1 0) planes of the *R*3 structure (Figure S4c, Supporting Information). Thus, the cubic morphology is shaped by the exposure of {1 1 0} crystallographic planes of the rhombohedral structure. The energy dispersive spectrometer analysis (EDS) was conducted on a single cube nanocrystal and the composition was determined to be Pd_44.1_Ni_42.9_P_13.0_, which is closer to the ideal composition of *R*3 nanocrystals (Table S1, Supporting Information).

After the leaching treatment, the crystalline nanoclusters can be selectively removed, as demonstrated by synchrotron X‐ray total scattering experiments (Figure S5a, Supporting Information). The total scattering function (i.e., S(*Q*)) curve of the pristine Pd‐Ni‐P MG exhibits the typical feature of an amorphous material, characterized by broad diffuse signals.^[^ ^18^, ^41^ ^]^ After the heating‐quenching treatment, as expected, sharp Bragg diffraction signals appear, fingerprinting the presence of dispersed crystalline nanoclusters. Remarkably, the S(*Q*) curve of the leached sample shows a significant reduction in the diffraction signals, indicating the successful removal of the nanocrystalline components. This selective leaching could also be evidenced by comparing the X‐ray diffraction (XRD) patterns of the samples at these three stages (Figure S5b, Supporting Information). The success of selective leaching is attributed to the tailored degree of atomic ordering, which results in varying chemical stabilities against corrosion. Figure S6a (Supporting Information) presents the photographs of the Pd‐Ni‐P samples at different treatment processes. The pristine Pd‐Ni‐P MG exhibits a metallic luster, which remains unchanged when directly subjected to the leaching, indicating the high corrosion resistance of the pristine MG. This is further confirmed by the scanning electron microscopy (SEM) image shown in Figure S6b (Supporting Information). However, when the heating‐quenching process is included, the surface of the sample turns black, suggesting that the selectivity in corrosion arises from the distinct structural characteristics between amorphous and nanocrystalline materials. It is well‐established that the MGs, lacking grain boundaries and characterized by a disordered atomic arrangement, ensure a uniform distribution of chemical composition. These structural characteristics inhibit the formation of local electrochemical cells, thus enhancing corrosion resistance.^[^ ^42^, ^43^ ^]^ So, the selective leaching method facilitates the removal of nanocrystalline regions while preserving the amorphous matrix, enabling the construction of a porous structure.

Figure 1d displays the SEM image of the sample after an 800 s leaching period, revealing uniformly distributed nanopores on the surface of the Pd‐Ni‐P MG. The thickness of the nanoporous region was about 3.33 µm. The corresponding TEM image confirms the amorphous structure and porous morphology of the leached MG (Figure S7, Supporting Information). Figure S8 (Supporting Information) shows the SEM images of the samples subjected to different leaching durations. At 200 s of leaching time, the cubic pore shape could be partially maintained (Figure S8a, Supporting Information). With prolonged leaching time, the average pore size gradually increases, and their shapes become more irregular. Meanwhile, the thickness of the porous region also grew to a depth of 8.35 µm (Figure S8d–S8f, Supporting Information). Based on the EDS results, the composition of the porous MGs was almost unchanged with increasing leaching time, but compared to the initial state samples, there was a decrease in Ni and Pd content and an increase in P content (Table S2, Supporting Information). This compositional change aligns well with the selective removal of the P‐poor rhombohedral nanoclusters.

To gain deeper insights into the local structure of the porous MG, scanning transmission electron microscopy equipped with a high‐angle annular dark field (HAADF‐STEM) was utilized. Figure S9a (Supporting Information) presents an atomic‐scale image of the nanoporous Pd‐Ni‐P MG, which consists of medium‐range‐ordered 6M‐TTP clusters with various orientations and degrees of distortion (Figure S9b–S9d, Supporting Information). The atomic pattern observed in the HAADF‐STEM image was simulated based on the Atom‐Match Programme^[^ ^38^ ^]^ (refer to Methods in the Supporting Information), which allowed for the automatic identification of six 6M‐TTP clusters with different orientations (Figure S9e–S9j, Supporting Information). The porous structure of the Pd‐Ni‐P MG was further characterized by 3D tomography reconstruction. A fragment of the porous MG was selected for the reconstruction, with the EDS mapping validating the presence of well‐defined pores and a homogeneous distribution of Pd, Ni, and P elements (Figure S10, Supporting Information). The HAADF‐STEM images for tomography reconstruction were captured at 2° intervals (see Video S1 (Supporting Information); and Figure 1e in which the five selected images are presented), and the frontal view of the reconstructed sample is presented in Figure 1f. By analyzing the cross‐sectional images taken at equal intervals along the *z*‐axis, we confirmed that the pores within the MG were uniformly distributed and interconnected. For better visualization, a subvolume cube was extracted for contract segmentation (Figure 1g). This process distinguished the high‐contrast region (i.e., MG matrix) from the low‐contrast region (i.e., pores), validating the internal continuity of both MG matrix and pores (see Videos S2 and S3, Supporting Information). Volumetric analysis quantified the porous volume as 9.78 nm^3^ and the solid volume as 3.54 nm^3^. These results demonstrate the successful fabrication of Pd‐Ni‐P MG using selective leaching, giving rise to a continuous, fracture‐free porous MG with high mechanical stability. This architecture increases specific surface area and active sits, facilitating electrolyte diffusion and enhancing reaction kinetics, ultimately benefitting electrochemical performance.

**Figure 2:**
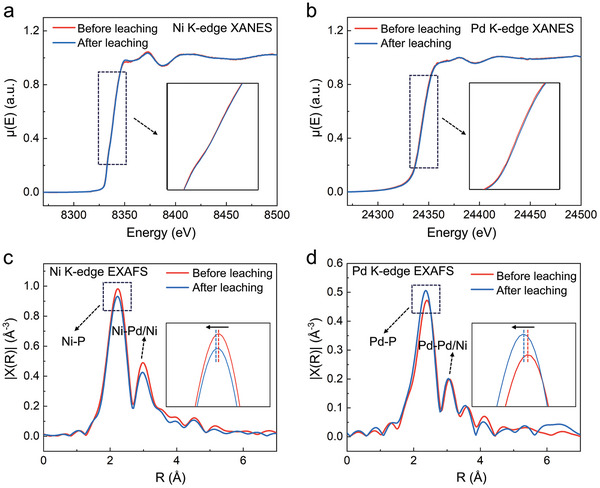
The XAFS of Pd‐Ni‐P samples before and after leaching. The XANES spectra of Ni‐K edge a) and Pd‐K edge b). The inset image shows a magnification of the absorption edges for the Ni‐K edge and Pd‐K edge, respectively. The EXAFS spectra of Ni‐K edge c) and Pd‐K edge d). The inset image shows a magnification of the Ni*─*P peak and the Pd*─*P peak, respectively.

The electronic structure of the nanoporous Pd‐Ni‐P MG was investigated using synchrotron X‐ray absorption near‐edge structure (XANES) experiments (**Figure** **2**a,b). No noticeable peak shift could be identified for the Ni and Pd K‐edges after the leaching process, indicating that the leaching process did not alter the valence states of the nanoporous Pd‐Ni‐P MG. However, the X‐ray photoelectron spectroscopy (XPS) experiments revealed an obvious decrease in the intensity of Ni^0^ peak (at ≈852.6 eV), while an increase in the intensity of Ni^2+^ peak (at ≈852.9 eV) upon leaching (Figure S11a, Supporting Information). Meanwhile, the Pd^0^ state was maintained during the entire leaching process (Figure S11b, Supporting Information). The increase in the Ni^2+^ content could be derived from the oxidation effect of HNO_3_ in the leaching solution,^[^ ^44^ ^]^ resulting in Ni*─*O terminated bonds on the surface of the MG, which could lower the activation energy of the electrochemical reaction in acid condition.^[^ ^45^ ^]^ The extended X‐ray absorption fine structure (EXAFS) analysis was employed to detect the local coordination environment of nanoporous Pd‐Ni‐P MG. As shown in Figure 2c,d, the first shells of the Ni and Pd K‐edge EXAFS curves, corresponding to the Ni*─*P and Pd*─*P bonds, respectively, revealed statistically shorter bond lengths after the leaching process. This suggests that the leached nanoclusters with higher atomic ordering may possess longer first‐shell distances, which is consistent with previous studies.^[^ ^23^, ^46^ ^]^ In addition, the first‐shell peak intensities of Ni and Pd EXAFSs exhibited opposite changing trends upon leaching—the Ni*─*P peak intensity decreases while the Pd*─*P peak intensity increases. The variation in peak intensities, which correlate with coordination numbers, suggests that the leached nanoclusters likely have a higher Ni content compared to the MG matrix.

**Figure 3:**
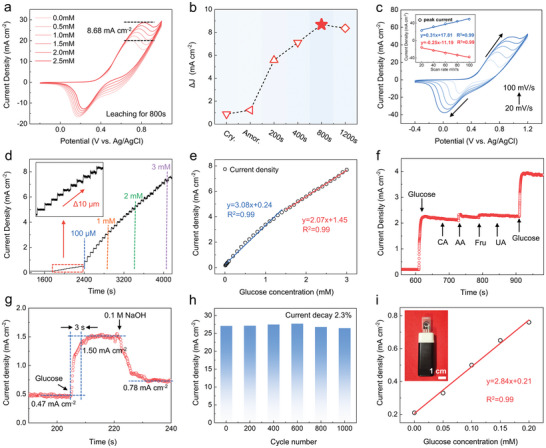
The electrochemical glucose sensing performance of nanoporous Pd‐Ni‐P MG. a) CV curves of nanoporous Pd‐Ni‐P MG after 800 s of leaching at different glucose concentrations. b) Variation of anodic peak current density for different samples. c) CV curves of the nanoporous Pd‐Ni‐P MG at different scan rates. The inset shows the linear relationship between the peak current density and scan rate. d) Typical amperometric curve of the nanoporous Pd‐Ni‐P MG after 800 s of leaching. The inset shows the amperometric current response under low concentrations of glucose. e) Linear fitting of current response data from (c). f) Interference tests of nanoporous Pd‐Ni‐P MG in the presence of CA, AA, Fru, and UA. g) Response‐recovery behavior. h) Change of anodic peak current density over 1000 CV cycles. i) Amperometric current response of the sensor device. The inset is a photograph of the fabricated sensor device.

A three‐electrode system was employed to evaluate the electrochemical glucose sensing performance of the nanoporous Pd‐Ni‐P MG. **Figure** **3**a compares the cyclic voltammetry (CV) results for various glucose concentrations. In the absence of glucose, a pair of redox peaks appeared at ≈0.69 and ≈0.18 V. With increasing glucose concentrations, a notable shift toward higher potentials in both oxidation and reduction peak positions was observed, likely due to the increased energy requirement for glucose diffusion on the electrode surface.^[^ ^8^ ^]^ Additionally, CV tests were conducted on the amorphous sample (prepared by melt‐spinning), crystalline sample (before leaching), and MG samples with different leaching periods (Figure S12, Supporting Information). The sensitivities were quantified based on the change in anodic peak current density (Δ*J*),^[^ ^47^ ^]^ with glucose concentrations ranging from 0 to 2.5 mm (Figure 3b). The 800‐s leached sample exhibited the highest Δ*J* value (i.e., ≈8.68 mA cm^−2^), corresponding to the highest glucose sensitivity. The enhanced electrochemical performance of pore‐engineered Pd‐Ni‐P MGs can be attributed to their unique structural properties and the benefits of pore engineering. The amorphous nature of these alloys results in a high density of catalytically active sites due to their lower atomic packing density and coordination unsaturation.^[^ ^48^, ^49^ ^]^ This structural advantage, combined with the metastable energy state from rapid quenching, allows for increased reactivity and corrosion resistance during electrochemical reactions. Additionally, the selective leaching process not only increases the specific surface area but also optimizes the porosity of the material, further enhancing the electrode's performance by maximizing the interaction between the electrode and electrolyte.^[^ ^20^, ^29^ ^]^ These combined factors position the pore‐engineered Pd‐Ni‐P MGs as superior candidates for electrochemical applications compared to traditional crystalline and amorphous materials.

As shown in Figure 3c, CV curves recorded at different scan rates revealed an increase in both anodic and cathodic peak currents and potentials. The linear relationship between peak currents and scan rates (Figure 3c inset) suggests a typical diffusion‐controlled process.^[^ ^50^ ^]^ Furthermore, chronoamperometry tests were conducted at an optimized working potential of 0.55 V (Figure S13, Supporting Information). As shown in Figures 3d and S14 (Supporting Information), the current density exhibited a distinct stepwise increase as the glucose concentration rose from 0 to 3 mm. Even with very small concentration changes, clear responses could be observed. By taking the steady‐state current values at the steps and performing linear fitting, we obtained the sensitivity of the sensor, as shown in Figures 3e and S14b (Supporting Information). The sample displayed a linear response in glucose concentration ranges of 0.001–1.5 mm and 1.5–3 mm, with average sensitivities of 3.19 and 1.91 mA mm⁻¹ cm⁻^2^, respectively. The detection limit is ≈2.94 µm, calculated using the limit of detection formula^[^ ^12^, ^51^ ^]^ (see details in Methods in the Supporting Information). Considering both sensitivity and linear range, our porous MG is among the best glucose‐sensing materials ever reported (Table S3, Supporting Information).

The potential of the prepared nanoporous Pd‐Ni‐P MG for practical sensing applications was further investigated through interference and stability tests. Figure 3f presents the interference test results. Glucose (0.5 mm) was initially added into the solution, followed by successive additions of interfering substances (0.1 mm), including citric acid (CA), ascorbic acid (AA), fructose (Fru), and uric acid (UA), and then additional glucose (0.5 mm). The current response to the interfering substances was negligible compared to the change induced by glucose, indicating the remarkable selectivity of nanoporous MG. The response recovery performance, shown in Figure 3g, demonstrates the capability of the sensor to react with the analyte and then return to the initial state after measurement. As glucose concentration increased, the current signal rapidly rose from 0.47 to 1.50 mA cm^−2^ within 3 s. Upon addition of NaOH solution to dilute the glucose concentration threefold, the current swiftly decreased to 0.78 mA cm^−2^, achieving a recovery rate of 96%. Furthermore, to evaluate the stability, 1000 cycles of CV testing were performed at a glucose concentration of 0.5 mm (Figure S15, Supporting Information). The results showed that the redox peaks remained almost unchanged, with the anodic peak current attenuating only 2.3%, demonstrating excellent cyclic stability (Figure 3h). Integrating this MG material with screen‐printed electrode (SPE), we successfully developed a miniature glucose sensor device (see Figure 3i inset; and Figure S16, Supporting Information). The glucose detection sensitivity of the device was 2.84 mA mm cm^−2^, which is close to the sensitivity value obtained from the electrochemical workstation (Figures 3i and S17a, Supporting Information). Meanwhile, SPE also has good stability (Figure S17b, Supporting Information).

It is well‐known that nonenzymatic electrochemical glucose sensors, while typically exhibiting high sensitivity, often suffer from relatively poor selectivity.^[^ ^52^ ^]^ This is due to the oxidations of many interfering species within the potential range of glucose oxidation, particularly in the materials containing transition metals.^[^ ^53^ ^]^ Meanwhile, noble metals are often impaired by the irreversibly adsorbed oxidation intermediates of glucose and chloride ions.^[^ ^54^ ^]^ However, the nanoporous Pd‐Ni‐P MGs have demonstrated remarkable anti‐interference capabilities and stability. This might be attributed to several factors: on the one hand, The presence of P element is the key to the preparation of amorphous alloys, which is the guarantee of excellent electrochemical properties and corrosion resistance. On the other hand, the low toxicity of Ni and the synergistic enhancement of oxidation current and anti‐interference by the combination of Pd and Ni.^[^ ^52^, ^55^, ^56^ ^]^ Furthermore, our XRD results indicate that the MG material can maintain its amorphous state after the reaction (Figure S18, Supporting Information), while the SEM and EDS analyses confirmed that the porous structure and composition also remained intact (Figure S19 and Table S3, Supporting Information). The high chemical stability of the amorphous nature is regarded to prevent the collapse of the nanoporous structure during prolonged reaction processes.

**Figure 4:**
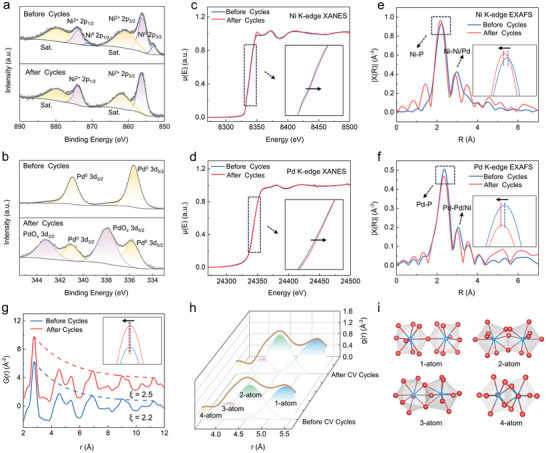
XPS spectra of a) Ni 2p and b) Pd 3d for the nanoporous Pd‐Ni‐P MG before and after 1000 CV cycles. The XANES spectra of Ni‐K edge c) and Pd‐K edge d). The inset image shows a magnification of the absorption edges for the Ni‐K edge and Pd‐K edge, respectively. The EXAFS spectra of Ni*─*K edge e) and Pd*─*K edge f). The inset image shows a magnification of the Ni*─*P peak and the Pd*─*P peak, respectively. g) The reduced pair distribution function G(*r*) patterns for the nanoporous Pd‐Ni‐P MGs before and after 1000 CV cycles. The inset is a magnification of the first shell h). The Gaussian fitting results of the second coordination shell in the g(*r*)s. i) The SRO can be connected in four different ways, characterized as 1‐atom, 2‐atom, 3‐atom, and 4‐atom cluster connections, where the SRO share one to four atoms.

To explore the elemental valence changes upon electrochemical reactions, XPS was conducted on the nanoporous Pd‐Ni‐P MGs before and after CV cycling. Before cycling, the Ni 2p_3/2_ XPS spectra exhibited peaks for Ni^0+^ at 852.93 eV and Ni^2+^ at 856.13 eV before CV cycling (**Figure** **4**a). After cycling, the Ni^0+^ peaks vanished, leaving only Ni^2+^ peaks at 856.38 eV. Previous studies have demonstrated that Ni reacts with electrolytes to form Ni(OH)_2_ during glucose detection. Then, Ni(OH)_2_ further reacts with OH^−^ to form NiO(OH), which subsequently reacts with glucose to produce glucolactone and generate an electrical signal.^[^ ^12^, ^57^, ^58^ ^]^ Regarding the Pd element, peaks of PdO*_x_* at 337.98 eV (3d_5/2_) and 343.28 eV (3d_3/2_) appeared after cycling, suggesting surface oxidation of partial Pd^0^ (Figure 4b). The XANES at the Ni K‐edge and Pd K‐edge further support the XPS results (Figure 4c,d). Both the Ni and Pd K‐edges shifted to higher energy after CV cycling, indicating increased valence states. Furthermore, the corresponding EXAFS data of Ni and Pd showed shortened bonds after cycling, especially the Ni*─*P bond length reduced by ≈0.1 Å. These findings suggest decreased distance between Pd and Ni active sites, effectively reducing diffusion distance for glucose and free OH radicals, promoting the transport of electroactive species.^[^ ^59^, ^60^ ^]^

The structural origin of the nanoporous Pd‐Ni‐P MG contributing to the high electrochemical stability was investigated by synchrotron X‐ray PDF analysis.^[^ ^41^ ^]^ Figure 4e displays the reduced G(*r*) profile of the nanoporous Pd‐Ni‐P MGs. The first shell, which corresponds to the bond length of the nearest (Pd, Ni)‐P, shows a slight contraction after cycling, which is consistent with the EXAFS results. In addition, we fitted the intensity decay trend from the first to the fifth G(*r*) peak with an exponential decay function (see Method in the Supporting Information).^[^ ^61^ ^]^ The generated exponential factor *ξ* for the cycled sample increased from 2.2 to 2.5, suggesting enhanced medium‐range ordering of the MG. Previous studies have demonstrated that high structural correlation in MGs can suppress crystallization, which is beneficial for electrochemical stability.^[^ ^23^ ^]^ Furthermore, the G(*r*) function was converted into g(*r*) with a function g(*r*) = G(r)/4π*rρ*_0_, where *ρ*_0_ represents the atomic number density.^[^ ^62^, ^63^ ^]^ The second peak of g(*r*), as shown in Figure 4h, reflects the connection modes between short‐range ordered clusters at medium‐range scales, including 1‐atom point‐sharing, 2‐atom edge‐sharing, 3‐atom‐face‐sharing, and 4‐atom interpenetrating connection modes (Figure 4i).^[^ ^64^, ^65^, ^66^ ^]^ Due to the isotropy of MGs, g(*r*) was fitted using Gaussian functions,^[^ ^62^ ^]^ with fitting parameters and fitting results listed in Tables S5 and S6 (Supporting Information), respectively. The results show that the as‐prepared nanoporous Pd‐Ni‐P MG primarily consisted of 1‐atom and 2‐atom connection modes, where a longer correlation length is expected.^[^ ^23^ ^]^ After the CV cycling, the medium‐range ordered structure remained almost unchanged, with only 2% of atomic clusters transforming from 3‐ and 4‐atom connection modes to 2‐atom and 1‐atom connection modes. This indicates that the topological stacking of the nanoporous Pd‐Ni‐P MG remains stable upon cycling.

Typically, MGs with electrochemical reactivity are reported to crystallize due to relaxation, often leading to performance degradation.^[^ ^67^, ^68^ ^]^ However, our prepared nanoporous Pd‐Ni‐P MG can maintain highly correlated medium‐range ordering throughout the reaction process. The stabilized amorphous structure can suppress the crystallization. Moreover, the connection modes of clusters are dominated by point‐sharing and edge‐sharing manners. These configurations result in a loosely packed atomic arrangement in the nanoporous Pd‐Ni‐P MG. The performance of catalysts is linked to their surface energy states.^[^ ^69^ ^]^ For metal catalysts, the atomic packing density directly affects the surface, and lower atomic packing densities result in higher surface energies.^[^ ^70^ ^]^ Furthermore, MGs are typically produced through rapid quenching methods, making them high‐energy metastable materials due to kinetic factors,^[^ ^71^ ^]^ which may also account for their high electrochemical activity.

## 3 Conclusion

In conclusion, we developed a novel selectively leaching method for the fabrication of nanoporous Pd‐Ni‐P MG, which displays excellent electrochemical glucose sensing performance. In this process, TTP polyhedrons are initially rearranged to form a 6M‐TTP cluster, then 6M‐TTP is further periodically stacked to form nanocrystals, and finally, nanoporous Pd‐Ni‐P MG is prepared based on the heterogeneity of nanoscale heterostructures. Local structural investigations reveal a decrease in the distance between active sites during electrochemical cycling, which optimizes electronic transport. Meanwhile, the stable MRO structure is beneficial to the maintenance of performance in glucose sensing. The present work provides a novel ordering/entropy modulation strategy for fabricating catalytic materials with promising excellent electrochemical performance. It offers a detailed explanation of the correlation between local structural order and electrochemical property.

## Conflict of Interest

The authors declare no conflict of interest.

## Supporting information



Supporting Information

Supplemental Video 1

Supplemental Video 1

Supplemental Video 3

## Data Availability

The data that support the findings of this study are available from the corresponding author upon reasonable request.
