# Supplementary material for: Tailoring Atomic Ordering Uniformity Enables Selectively Leached Nanoporous Pd‐Ni‐P Metallic Glass for Enhanced Glucose Sensing
Source: Adv Sci (Weinh). 2024 Nov 5;11(48):2408816. doi: 10.1002/advs.202408816 (PMC11672269; doi:10.1002/advs.202408816)
Supplement: Supplementary file 1 — Supporting Information [file ADVS-11-2408816-s002.docx]

**Supporting Information**

**Tailoring Atomic Ordering Uniformity Enables Selectively Leached Nanoporous Pd-Ni-P Metallic Glass for Enhanced Glucose Sensing**

Yu Lou^1,#^, Jian Li^2,#^, Zhongzheng Yao^1,#^, Zhenduo Wu^3,4^, Huiqiang Ying^1^, Lan Tan^1^, Sinan Liu^1^, Jianrong Zeng^5,6^, Ruohan Yu^7*^, Hong Liu^8^, Xun-Li Wang^4^, He Zhu^1*^, Si Lan^1,4*^

^1^*Herbert Gleiter Institute of Nanoscience, School of Materials Science and Engineering, Nanjing University of Science and Technology, Nanjing 210094, China*

^2^*School of Bioscience and Technology, Chengdu Medical College*

*^3^City University of Hong Kong (Dongguan), Dongguan, 523000, China*

*^4^Department of Physics, City University of Hong Kong, 83 Tat Chee Avenue, Kowloon, Hong Kong SAR, China*

^5^*Shanghai Synchrotron Radiation Facility, Shanghai Advanced Research Institute, Chinese Academy of Sciences, 201204 Shanghai, P. R. China.*

^6^*Shanghai Institute of Applied Physics, Chinese Academy of Sciences, 201800 Shanghai, P. R. China*

^7^*State Key Laboratory of Advanced Technology for Materials Synthesis and Processing, Wuhan University of Technology, Wuhan 430070, P. R. China*

^8^*State Key Laboratory of Bioelectronics, School of Biological Science and Medical Engineering, Southeast University, Nanjing 210096, China*

**Contents**

1. **Experimental Section**

**Synthesis of Pd-Ni-P MGs** **(contain crystalline cube phase).**

Pd-Ni-P MGs (containing cube phase) was synthesized using a combined induction arc melting method and fluxing method. The Pd-Ni-P ingot was melted in an induction furnace and then fluxed with B_2_O_3_ in a quartz tube at a high temperature (1472 K) for 2 hours. After quenching in the air for 85 s (about 623 K), the ingot was transferred to a furnace at 623 K and treated at a constant temperature for 80 mins, followed immediately by water quenching.

**Strategy for the preparation of nanoporous structures.**

Due to the significant difference in corrosion resistance, a selective corrosion method strategy was proposed for the preparation of micro-nano porous structures. leaching treatment of heat-treated Pd-Ni-P MG (contain cube phase), which was pre-cut in diameter of about 3 mm and thickness of ~300 μm slice, was carried out for different times (200s, 400s, 800s, and 1200s) by using nitric and acetic acid solutions with a volume ratio of 1:1.

**Synthesis of Pd-Ni-P amorphous ribbon.**

As a comparison, Pd_41.25_Ni_41.25_P_17.5_ amorphous ribbons were also prepared using the spindle-type copper roll melt-spinning technique under an argon atmosphere, with a wheel speed of ~ 4000 r/min. The resulting ribbons have a width of ~3 mm and a thickness of ~45 µm.

**Conventional characterization**

The obtained sample was characterized by room temperature (RT) X-ray powder diffraction. A diffractometer (Bruker-AXS D8 Advance, Cu Kα radiation, λ =1.5418 Å) with a Bragg-Brentano reflection geometry was used. The diffraction patterns were recorded by continuous scanning in the 2θ range of 30–60° at an interval of 0.02°. DSC experiment was performed on a Netzsch DSC 404 F3 using Al crucibles in a high–purity Ar atmosphere with a scanning rate of 20 K min. The sample mass in DSC experiment is ~20 mg. The morphologies of the micro-nano porous structures were observed using a field emission scanning electron microscopy (FESEM, JSM-7800F PRIME). The transmission electron microscopy (TEM) samples were prepared using the following procedure: Initially, a thin slice of the material was meticulously mechanically ground down to achieve a thickness of ~10 μm. Subsequently, the resulting thin foil was subjected to electropolishing in an aqueous electrolyte solution containing 16% perchloric acid, 42% methanol, and 42% acetic acid. The electropolishing process was carried out at a temperature of ~-40 °C using a twin-jet polishing system. For Nanoporous Pd-Ni-P, the sample is first ground and leached, then placed in ethanol for 20 minutes for ultrasonic vibration, and then left for 10 minutes to take the supernatant and drop it into the ultra-thin carbon net. The transmission electron microscopy (TEM) was performed with FEI Tecnai 12 TEM at ~ 120 kV. X-ray photoelectron spectrum patterns were collected on the thermo scientific K-Alpha (Al Kα radiation, hv=1486.6 eV). Elemental fine structure spectra were scanned with a flux energy of 50 eV in steps of 0.1 eV, and charges were corrected using C1s=284.80eV binding energy as the energy standard.

**The three-dimensional (3D) tomography reconstruction**

The TEM/STEM images, EDS spectra, and electron tomography experiments were conducted using a state-of-the-art CEOS probe-corrected FEI Themis TEM instrument. The instrument operated at an electron-accelerating voltage of 300 kV, ensuring high-resolution imaging capabilities. A Gatan image filter spectrometer was utilized for data collection.The probe convergence angle was set to 17.8 mrad, and a probe current of approximately 10 pA was employed for both STEM imaging and EDS acquisition. A Fischione tomography holder with a tilt range of -80° to +80° was utilized to perform electron tomography,. The resulting tomographic data was reconstructed using Thermo Fisher 3D Inspect software and subsequently visualized using Avizo.

**Atom probe tomography (APT) measurements**

Samples for APT measurements were prepared by two-stage electropolishing, with the first stage using an electrolyte containing 30 vol% perchloric acid in acetic acid at 15 V and the second using 5 vol% perchloric acid in 2-butoxyethanol solution at 20 V. APT experiments were performed using a local electrode atom probe (LEAP4000X Si) with a UV laser pulse repetition rate of 200 kHz, pulsing laser energy of 0.4 pJ, a target evaporation rate of 0.5% and a specimen temperature of ~20 K. Reconstruction and visualization of the APT data were performed using the CAMECA Visualization and Analysis Software (IVAS v.3.6.8) package.

**Atom-Match Programme**

A Python program (version 3.6.9) was designed for the initial processing of HAADF images^[1]^. This software identifies the orientation of the 6M-TTP cluster within a cubic phase by aligning calculated HAADF-STEM image projections with actual experimental images. Three primary symmetry orientations of the 6M-TTP cluster, namely [111], [110], and [100], are projected and used for this comparison. The program operates through the following procedures:

1.Image Processing: In the HAADF-STEM images, areas brighter than a preset threshold are recognized as atoms. All other regions are considered as interatomic spaces.

2.Model Matching: Projections based on these orientations are manipulated by rotating (3 degrees per step) and translating (1 pixel per step) to align with the atomic positions in the experimental HAADF-STEM images. Each projection's pattern is compared against the processed image.

3.Matching Condition: Patterns that achieve a similarity index over 70% are preliminarily chosen for further detailed analysis. This similarity index is calculated by the ratio of the number of atoms in the calculated pattern that coincide within the atomic areas of the processed image to the total number of atoms in the model

**Synchrotron high-energy X-ray total scattering**

Synchrotron high-energy X-ray total scattering measurements for PDF analysis were performed at the beamline 11-ID-C (Advanced Photon Source, Argonne National Laboratory). The high-energy X-rays with a beam size of 500 µm × 500 µm and a wavelength of 0.1173 Å were utilized for data collection using a transmission geometry. Two-dimensional diffraction patterns were obtained using a Perkin Elmer amorphous silicon detector. With Fit2D and PDFgetX2^[2]^, the static structure factor S(Q) was derived from the scattering data after masking bad pixels, integrating images, subtracting the appropriate background, and correcting for oblique incidence, absorption, multiple scattering, fluorescence, Compton scattering and Laue correction. The reduced PDF G(r) was obtained from the Fourier transform of S(Q) according to the following equation^[3]^:

$$\begin{aligned} \text{G}\left( \text{r} \right)\text{ = }\left( \frac{\text{2}}{\text{π}} \right)\text{ × }\int_{\text{0}}^{\text{Q}_{\text{max}}} \text{Q}\left[ \text{S}\left( \text{Q} \right)\text{-1} \right]\sin\left( \text{Qr} \right)\text{d}\text{Q}\#\left( \text{1} \right) \end{aligned}$$

The PDF:

$$\begin{aligned} \text{g}\left( \text{r} \right)\text{ = 1 + }\text{G}\text{(}\text{r}\text{)}\text{ }\text{/}\text{ }\text{4}\text{πr}\text{ρ}_{\text{0}}\#\left( \text{2} \right) \end{aligned}$$

was derived from *G*(*r*) where *ρ_0_* is the number density. The *g*(*r*) contains more structural information.

The decay function:

$$\begin{aligned} f\left( \text{r} \right)\text{ = }\text{}\#\left( \text{3} \right) \end{aligned}$$

**PDF analysis**

In order to extract information about second-nearest-neighbour parts from the first and second shells, we transformed *G*(*r*) into the PDF *g*(*r*), since the profiles are closer to a Gaussian function for isotropic amorphous system.$W_{ij}$ is the weight of the atomic pair^[4]^: $\begin{aligned} \text{ω}_{\text{ij}}\text{ = 2}\text{c}_{\text{i}}\text{c}_{\text{j}}\text{b}_{\text{i}}\text{b}_{\text{j}}\text{/}\text{b}^{\text{2}}\text{(}\text{i }\text{≠ }\text{j}\text{) or }\text{c}_{\text{i}}\text{b}_{\text{i}}\text{/}\text{b}^{\text{2}}\text{(}\text{i }\text{=}\text{ j}\text{) }\#\left( \text{4} \right) \end{aligned}$

$$\begin{aligned} \text{b}\text{ = }\sum_{\text{i}} \text{c}_{\text{i}}\text{b}_{\text{i}}\#\left( \text{5} \right) \end{aligned}$$

where *i* and *j* are the *i*th and *j*th atomic species, *w_ij_* is the weight of the *i*–*j* pair, *c_i_* (*c_j_*) is the atom fraction, *b_i_* (*b_j_*) is the atom scattering factor and *b* is the average scattering length. $R_{ij}^{0}$ is the sum of atomic radii of different atomic pair (as shown in Table S3). The average bond length (first peak position) was R = 2.62Å according to the weighted bond length of the coordination atom. The cluster centre-to-centre distances of the four polyhedron connection modes, 1-atom, 2-atom, 3-atom and 4-atom connection, were calculated to be 2R, √3R, √8 /3R and √2R, respectively^[5]^.

**X-ray Adsorption Fine Structure analysis**

X-ray Adsorption Fine Structure (XAFS) patterns were collected at the BL13SSW beamline of the Shanghai Synchrotron Radiation Facility (SSRF). The center energy of the radiated X-rays was 50.00 keV. Data were collected at the K-edges of Pd, and Ni in the fluorescence mode. The Pd and Ni scans were run at the Ni-Ni K-edge absorption of 24350 eV and 8333 eV. Athena was used to process the X-ray absorption curves by energy shift, phase correction, normalized and obtain the R-space data of Ni and Pd with an FT range of 3-9.5 Å^−1^ and 3-10 Å^−1^. All spectral data were collected at room temperature in transmission mode.

**Electrocatalytic measurements**

The electrochemical measurements were conducted using a standard three-electrode system at a temperature of approximately 298 K, employing an electrochemical workstation (CHI 660E). A 0.1 M NaOH solution was used as the electrolyte for the non-enzymatic glucose sensor. First, the Pd-Ni-P electrodes with porous structure prepared above were taken and glued to polished and cleaned copper wire surface by conductive silver paste and insulated, and then the electrodes were placed in the air to dry. Within the three-electrode system, the Pd–Ni–P electrode, Pt film, and Ag/AgCl electrode were used as the working electrode, counter electrode, and reference electrode, respectively. Cyclic voltammetry (CV) was performed in glucose concentrations ranging from 0 to 2.5 mM with a scan rate of 20 mV/s, and at a fixed glucose concentration of 1 mM with scan rates ranging from 20 to 100 mV/s. The potential range was controlled from -0.4 to 1.2 V vs Ag/AgCl. The chronoamperometry was evaluated at 0.55 V (vs. Ag/AgCl) under magnetic agitation.

Sensitivity was calculated as the slope of the glucose sensor calibration curve. The limit of detection (Lod) is the lowest detected concentration with a peak height greater than or equal to the mean of the blank sample (no analyte) plus three standard deviations of the blank. Lod was calculated according to the following equation^[6,7]^:

$$\begin{aligned} \text{Lod}\text{ }\text{=}\text{ }\frac{\text{3}\text{δ}}{\text{S}} \#\left( 6 \right) \end{aligned}$$

where $\text{δ}$ is the standard deviation of blank samples in repeated examination, and S represents the slope of the calibration curve.

**A miniature glucose sensor device**

Firstly, The nanoporous Pd-Ni-P MG are affixed to the surface of the screen-printed electrodes (SPE) using conductive silver paste, and the surroundings are sealed with insulating adhesive to prevent interference from the conductive silver paste during testing. The electrochemical experiments were conducted on a miniature sensor device (SHENZHEN REFRESH BIOSENSING TECHNOLOGY CO., LTD,BIOSYS-P15E Max).The nanoporous Pd-Ni-P MG SPE was inserted into the plug remote of the sensor, and the device and a phone were connected via Bluetooth. The test results will be displayed on the phone.

1. **The Supplementary Figures and Tables.**

**
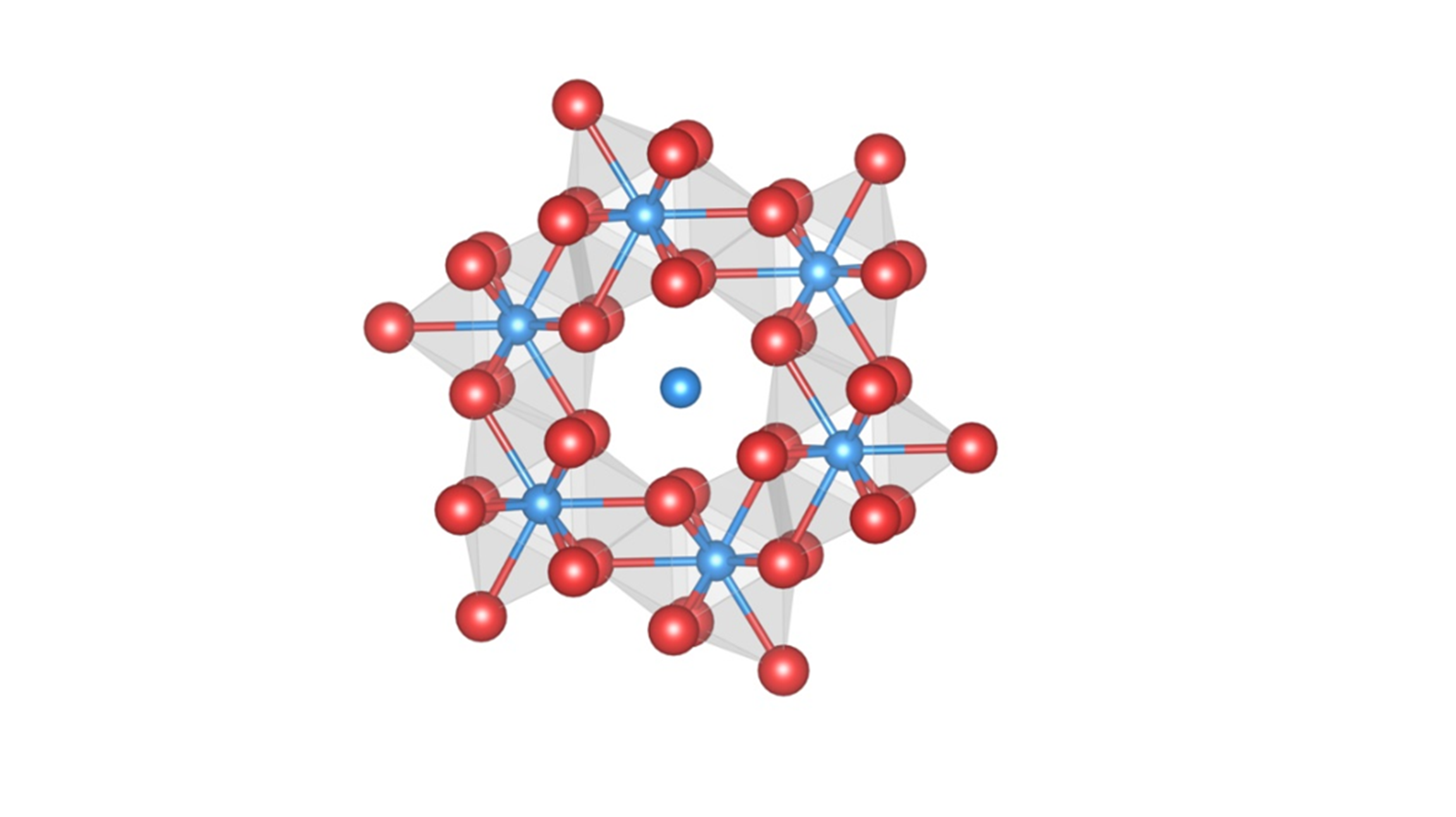
**

**Fig. S1** The schematic diagrams of the construction of the 6M-TTP cluster.


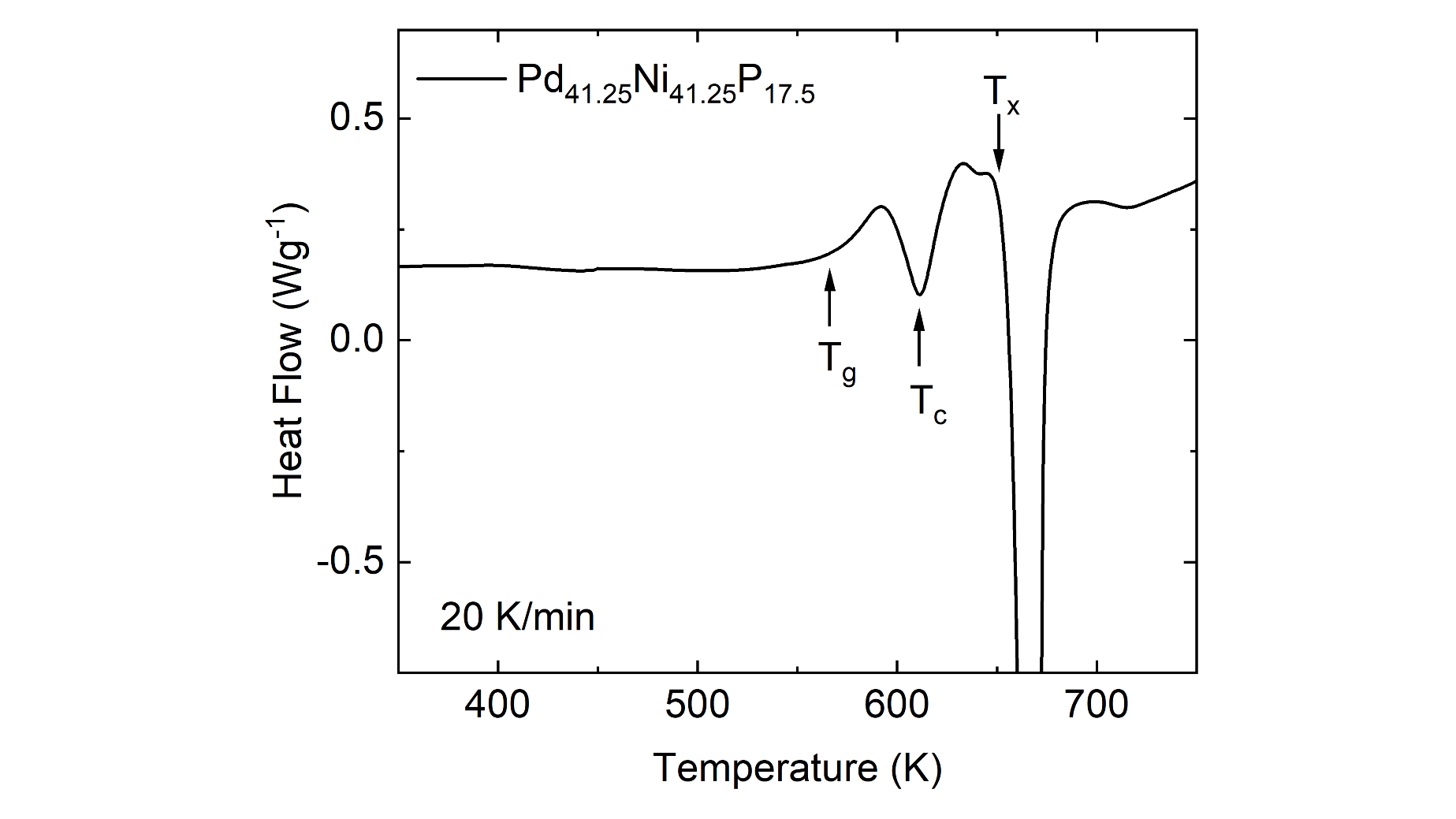


**Fig. S2** The DSC curve for Pd_41.25_Ni_41.25_P_17.5_ MGs at a heating rate of 20 K min^-1^ showing T_g_ (the glass transition temperature), T_c_ (the anomalous exothermal temperature) and T_x_ (the crystalline transition temperatures).

Note: Pd_41.25_Ni_41.25_P_17.5_ MGs is a good model system, showing fascinating thermal behavior during heating. It has been confirmed that reentrant will occur in the supercooled liquid phase region over the cold liquid phase transition at Tc, and its internal microscopic mechanism is the liquid-liquid phase change caused by the evolution of medium-range ordered structure^[8]^. In addition, the Pd_41.25_Ni_41.25_P_17.5_ MGs will precipitate the nano-metastable phase before heating to the final crystal^[1,9]^. Therefore, we choose heat treatment in the temperature range of the supercooled liquid phase to precipitate the nano-metastable phase.


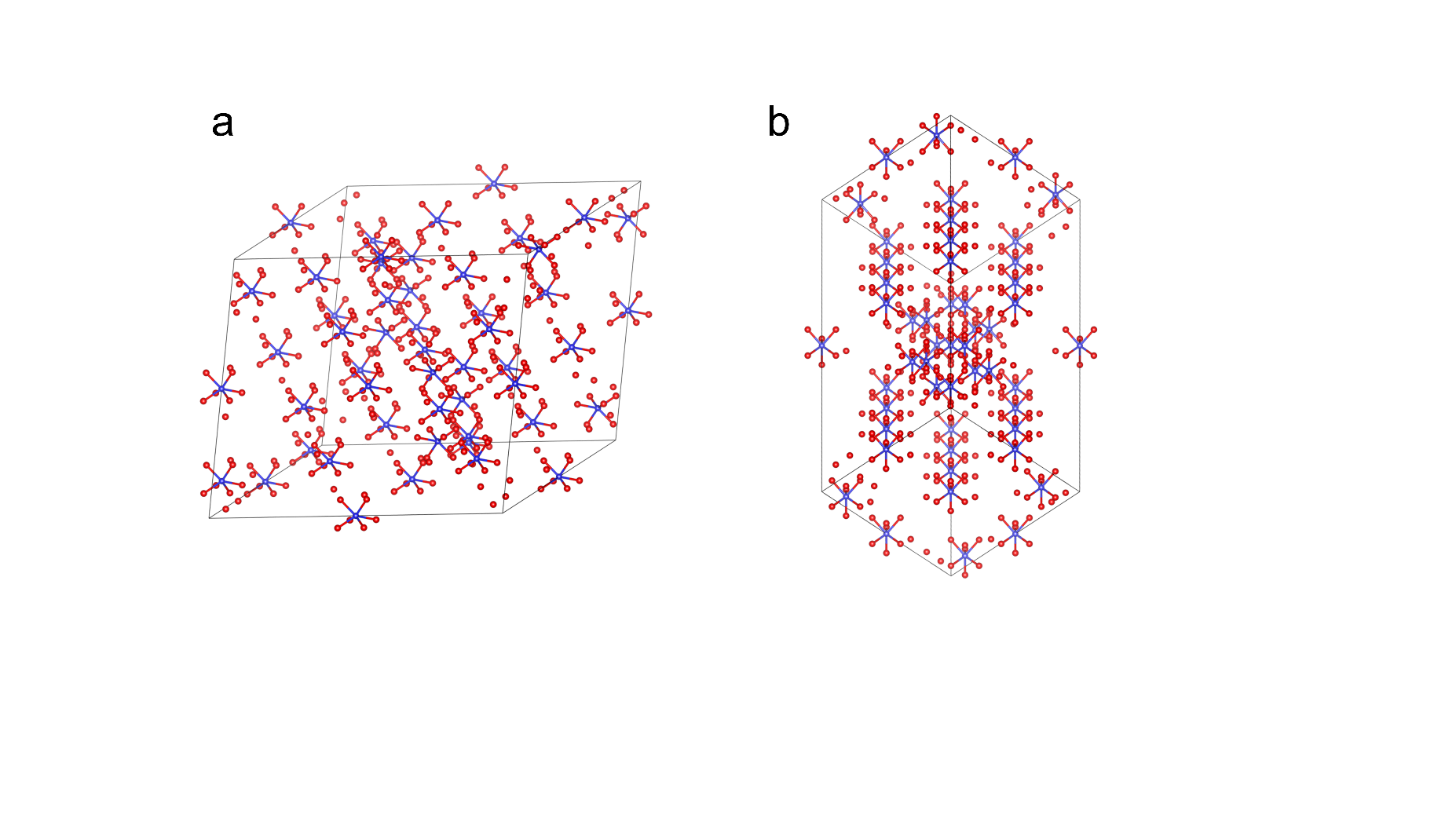


**Fig. S3** The rhombohedral R3 symmetric manner.


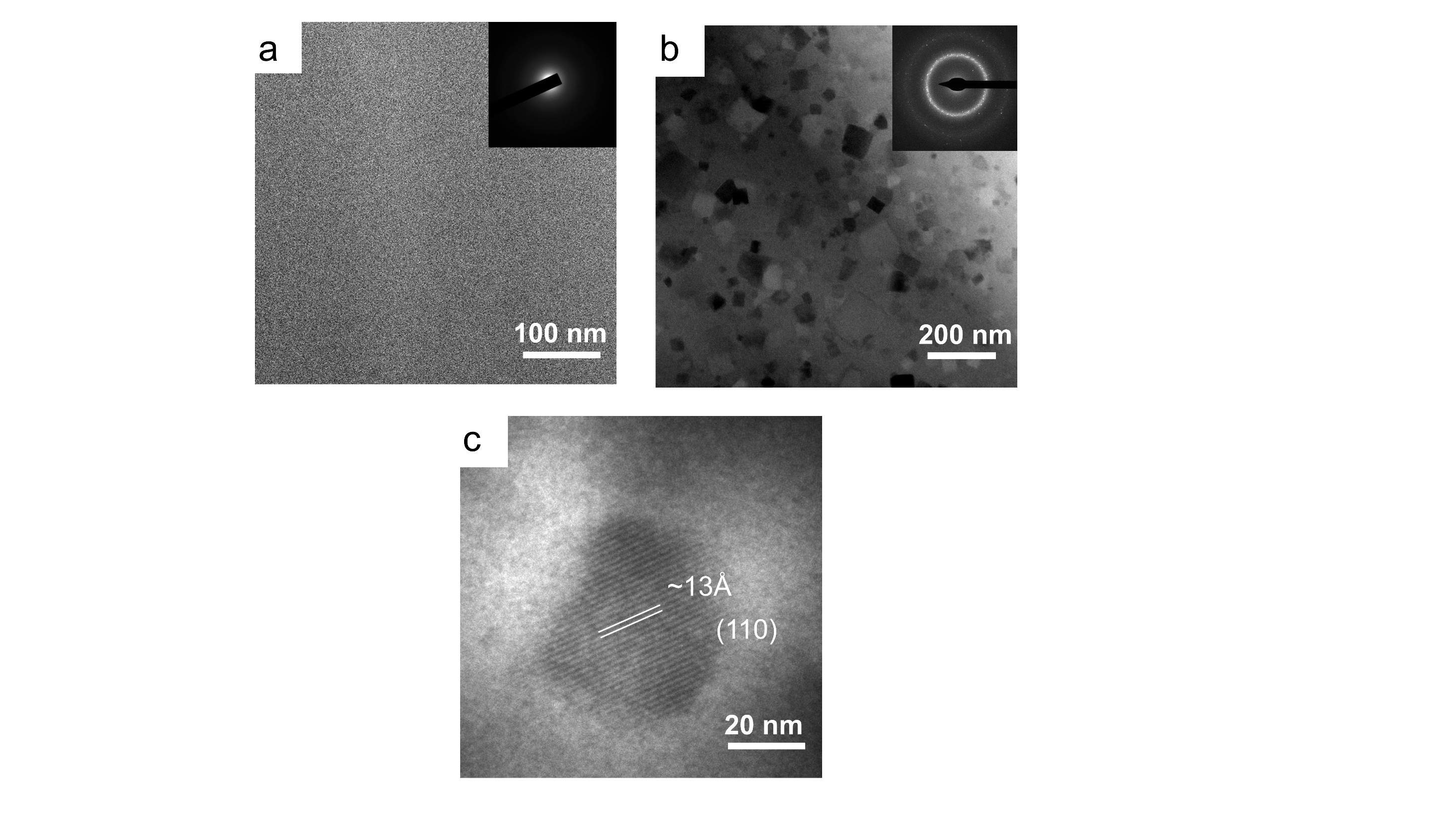


**Fig. S4** The TEM image of heating-treat Pd-Ni-P : (a) The as-prepared sample and (b) annealing for 80 min, the inset is the corresponding selected area electron diffraction pattern. (c) The HR-TEM image for the cube phase.


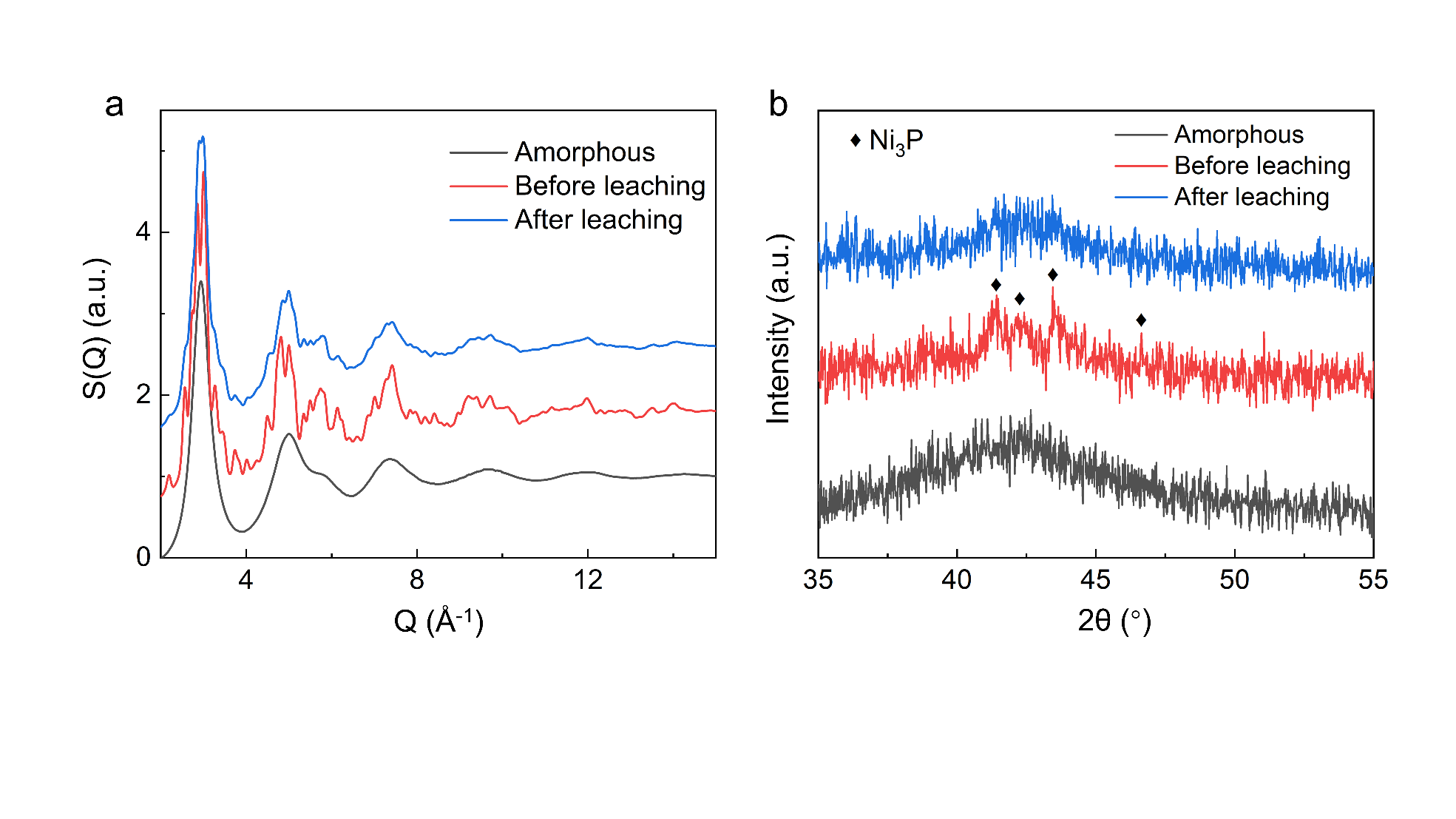


**Fig. S5** The S(Q) pattern (a) and XRD patterns (b) of amorphous sample prepared by melt-spinning, as well as samples before and after leaching. The diffraction peaks observed in the before-leaching pattern correspond to the Bragg peaks of the Ni3P crystalline phase.

Note:

The amorphous Pd-Ni-P sample exhibits a broad diffraction peak at around 42 degrees, characteristic of the amorphous nature. After the heating-quenching treatment, the Pd-Ni-P sample (before leaching) crystallizes, as evidenced by the appearance of sharp diffraction peaks corresponding to the Ni_3_P crystalline phase. Following the leaching process, the XRD pattern shows a significant reduction of these diffraction signals, indicating the successful removal of the nanocrystalline components.


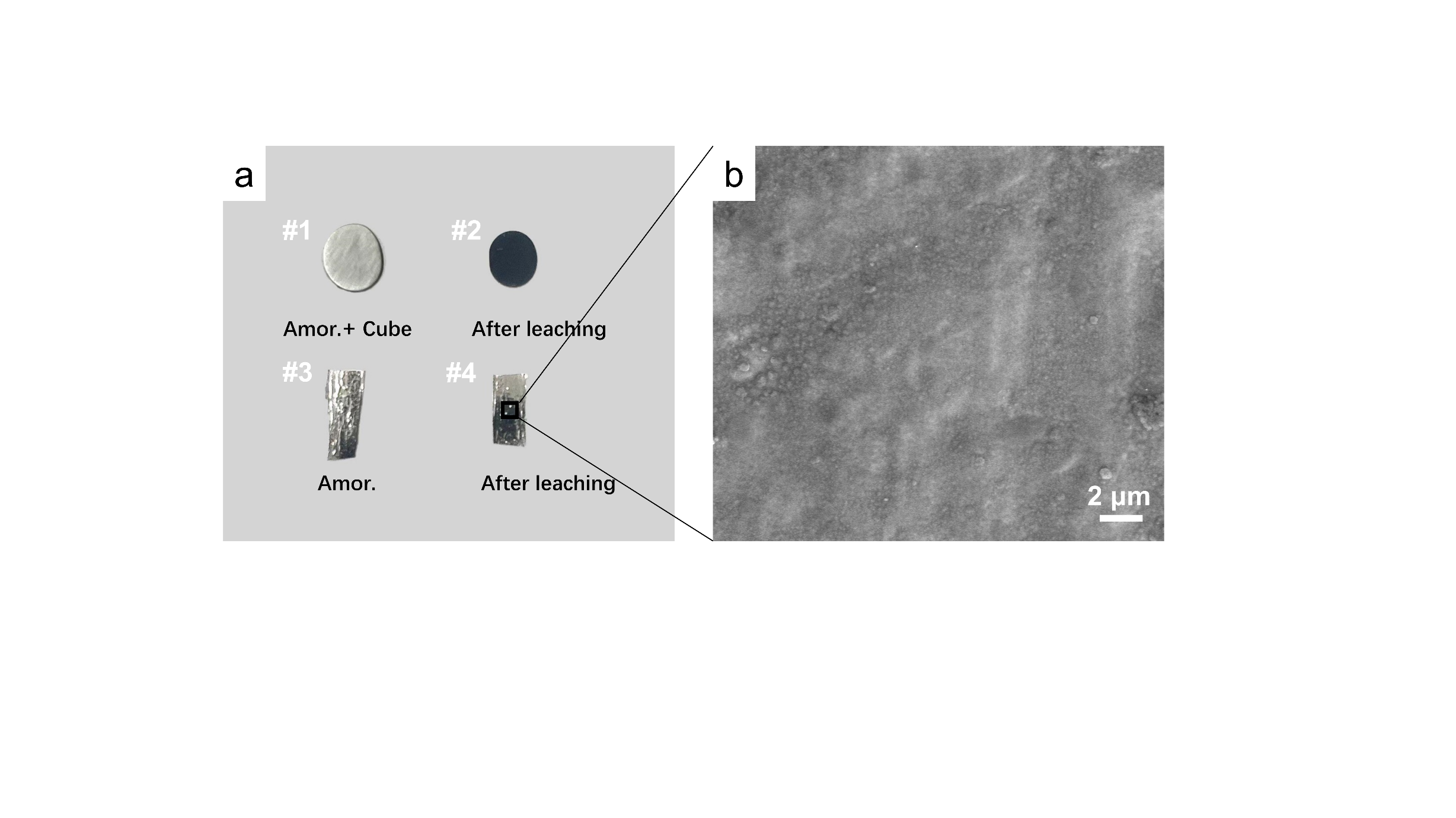


**Fig. S6** (a) The Pd-Ni-P samples in different states: the heat-treated samples (#1), the heat-treat samples after an 800-second leaching period (#2), the amorphous samples prepared by melt-spinning (#3) and the amorphous samples prepared by melt-spinning after an 800-second leaching period (#4). (b) The SEM image of the amorphous samples after an 800-second leaching period.


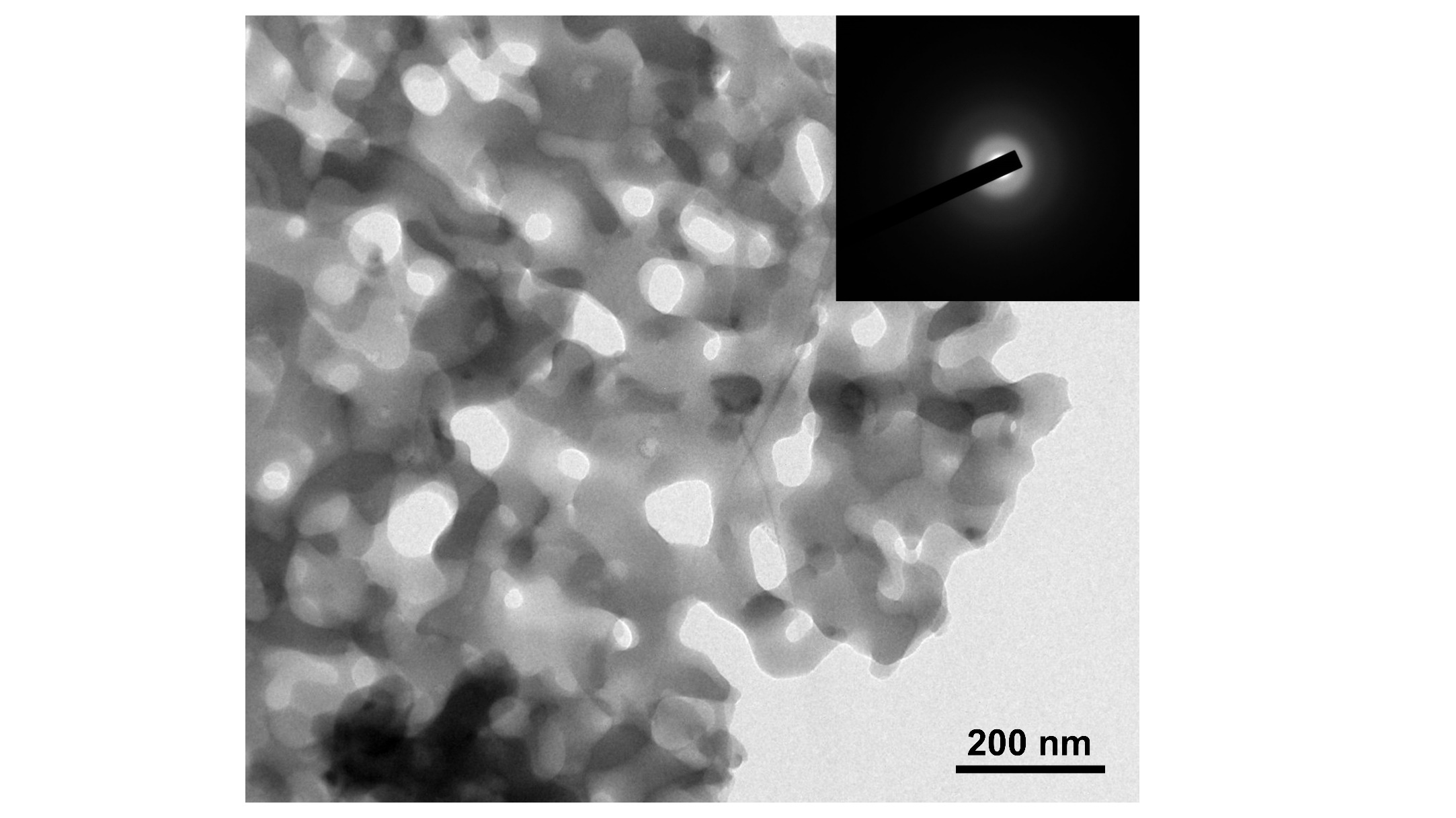


**Fig. S7** The TEM bright-field image for the nanoporous Pd-Ni-P MG after leaching. The inset is the corresponding selected area electron diffraction pattern.


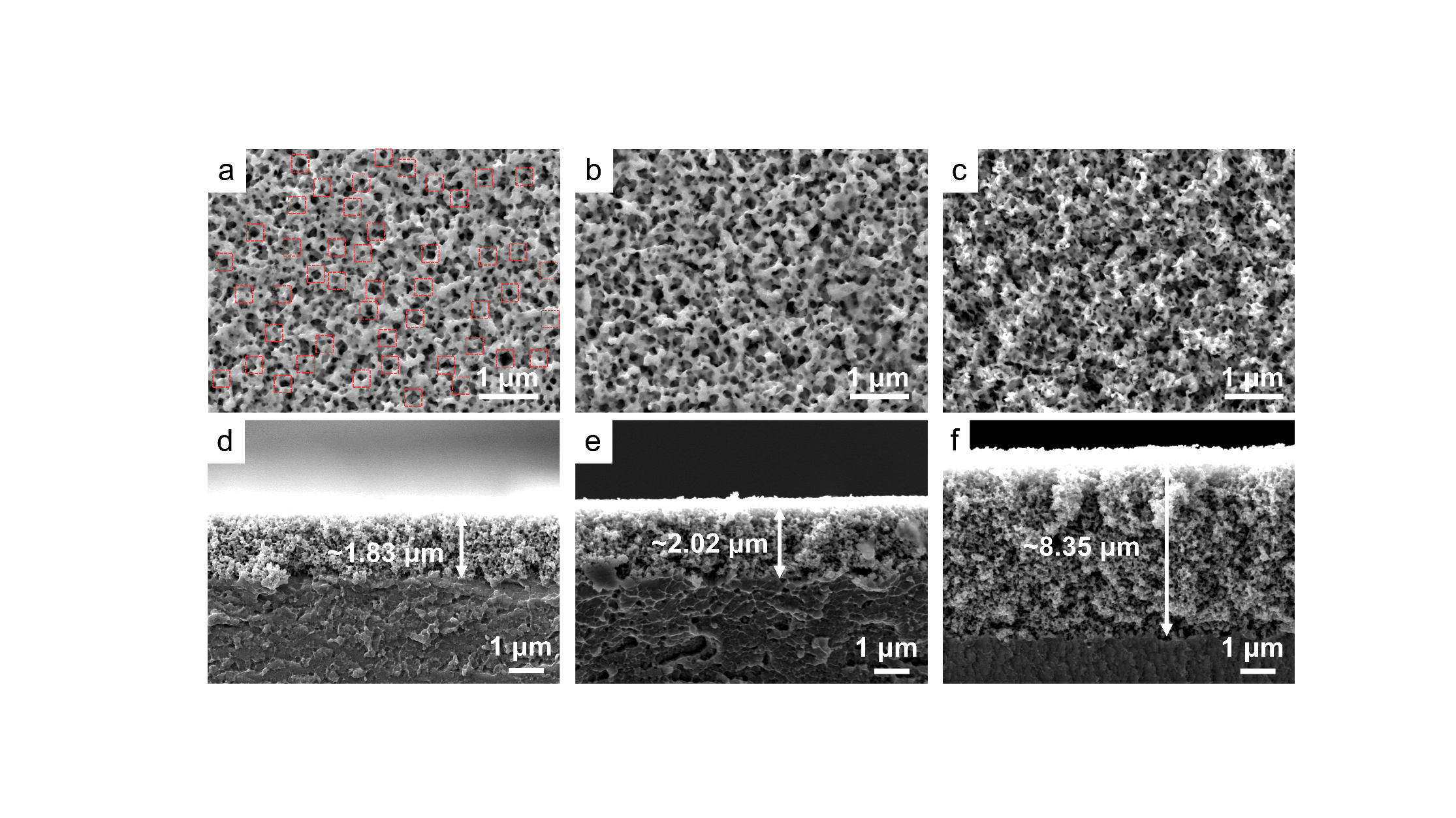


**Fig. S8** (a)-(c)The SEM image of the sample after 200,400,1200 seconds of leaching, respectively. (d)-(e) the corresponding corrosion thickness image of (a)-(c).


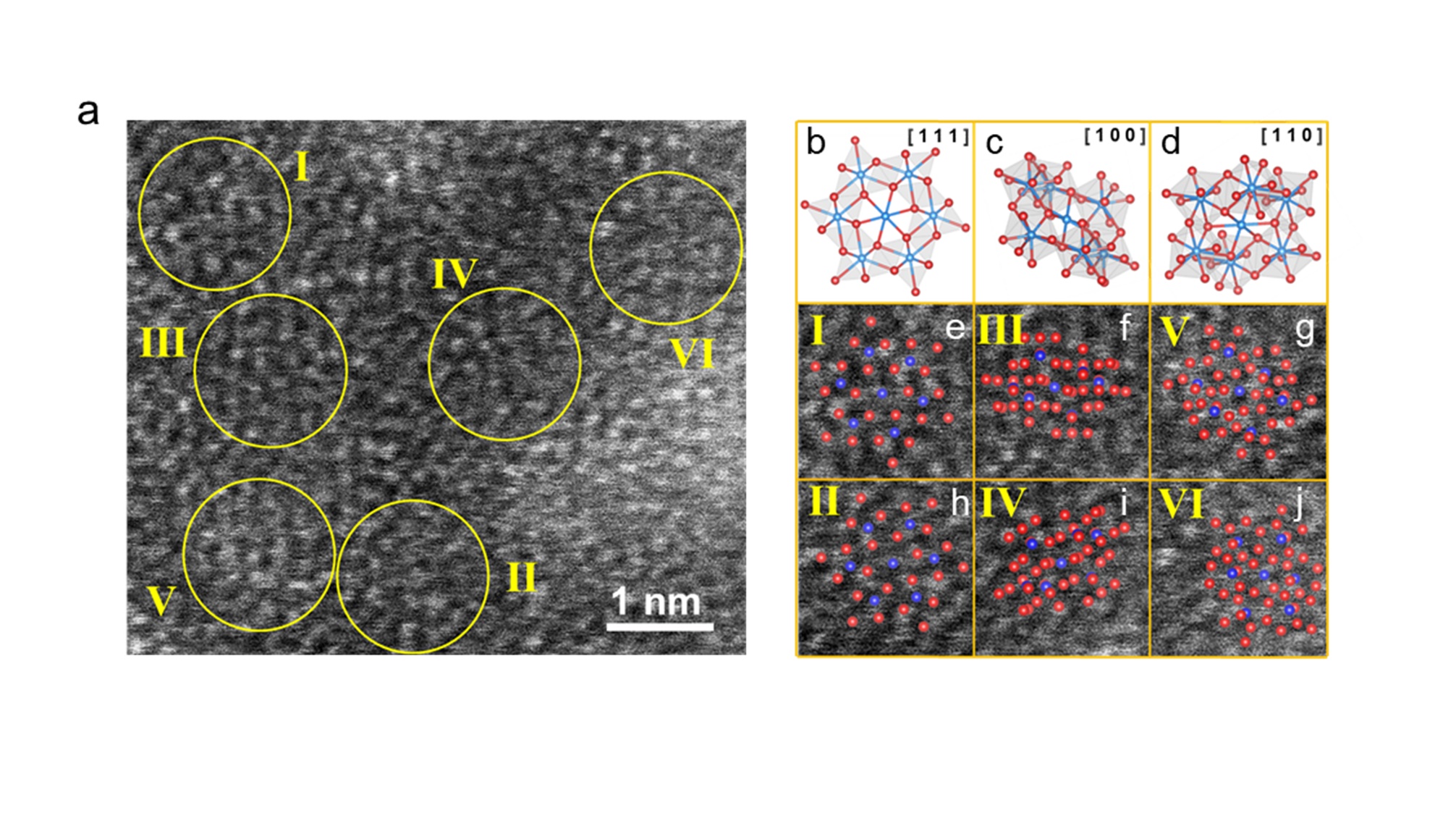


**Fig. S9** (a) The HAADF-STEM image of nanoporous Pd-Ni-P MG. (e) - (j) The cluster matching of nanoporous Pd-Ni-P MGs on the basis of the model 6M-TTP cluster [111] (b), [100] (c) and [110] (d) orientation.


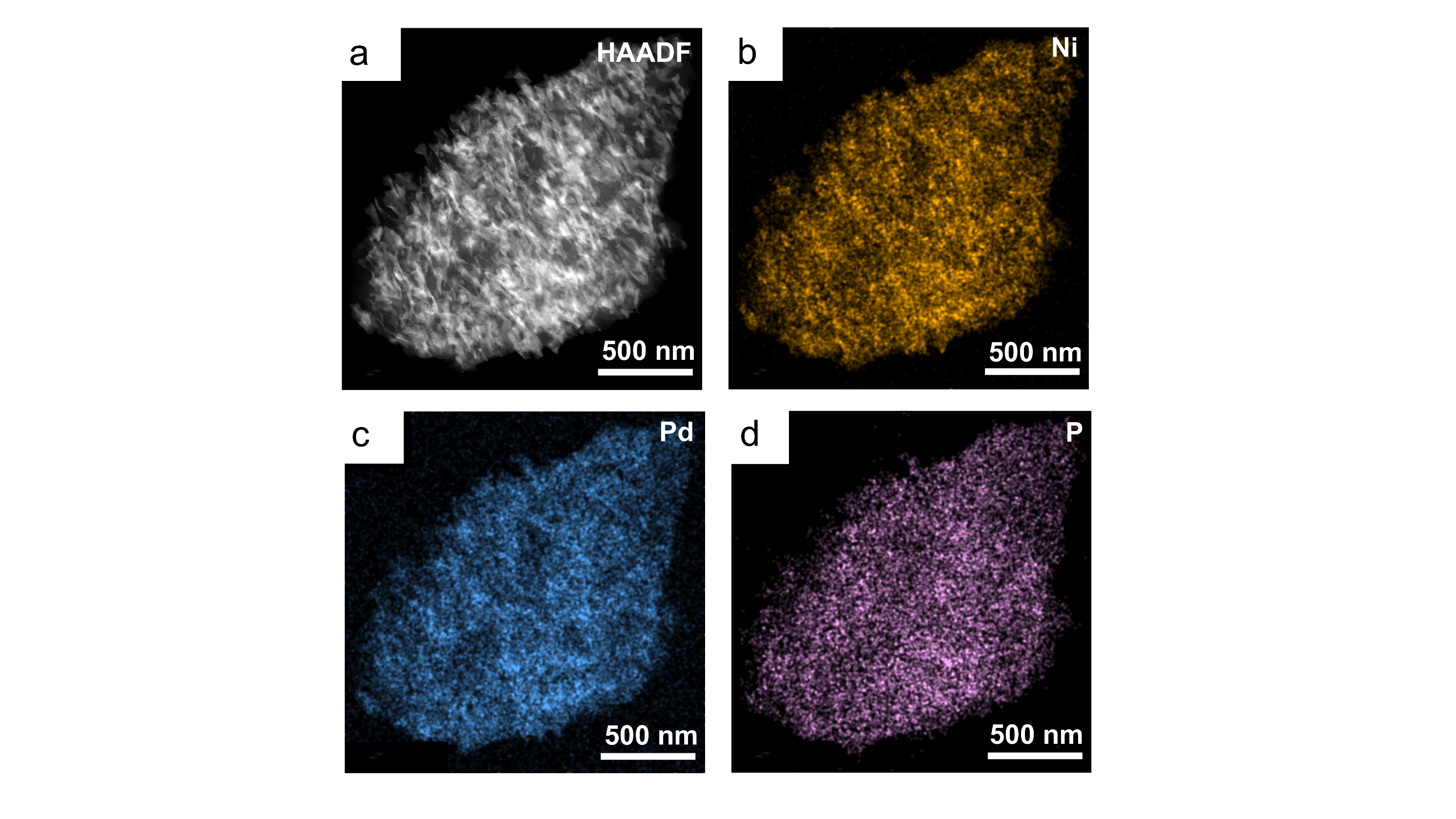


**Fig. S10** (a) The HADDF-STEM of nanoporous Pd-Ni-P MG and (b)-(d) corresponding EDS mapping images of Ni element, Pd element, and P element.


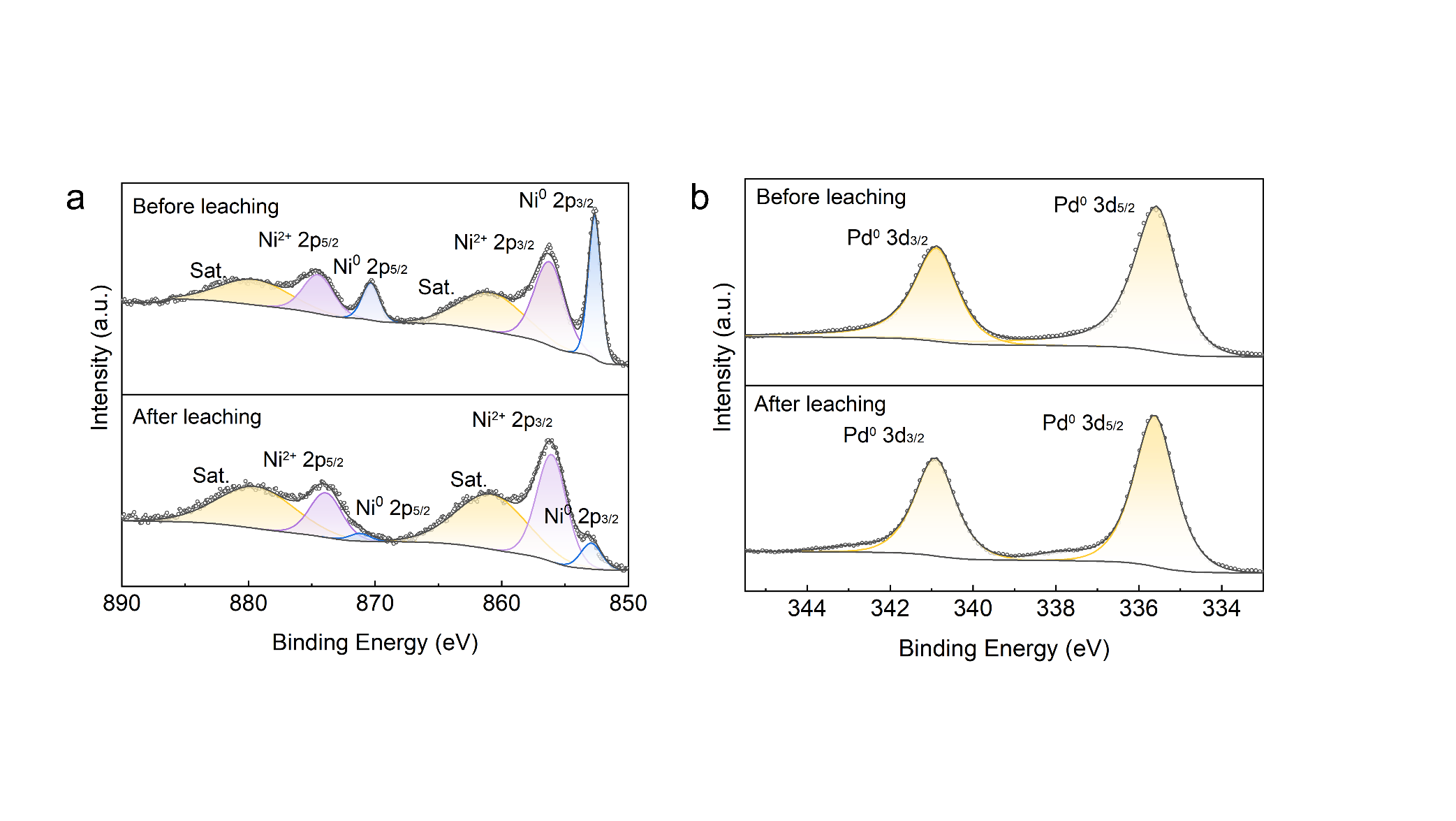


**Fig. S11** The XPS spectra of (a) Ni 2p, (b) Pd 3d for the Pd-Ni-P samples before and after leaching.


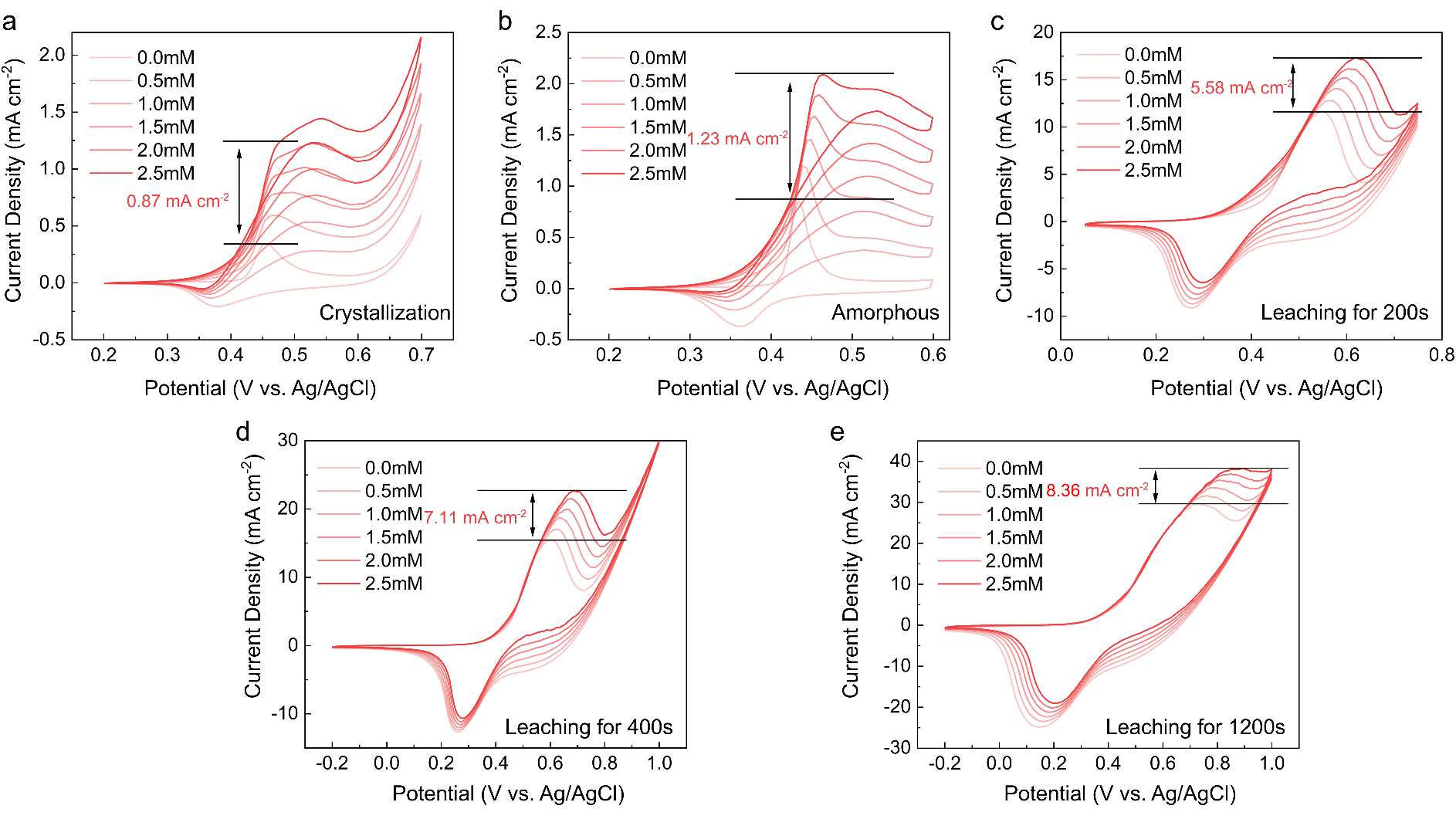


**Fig. S12** The CV curves for (a) crystalline, (b) amorphous and (c-e) leached for different durations of Pd-Ni-P at different glucose concentrations.


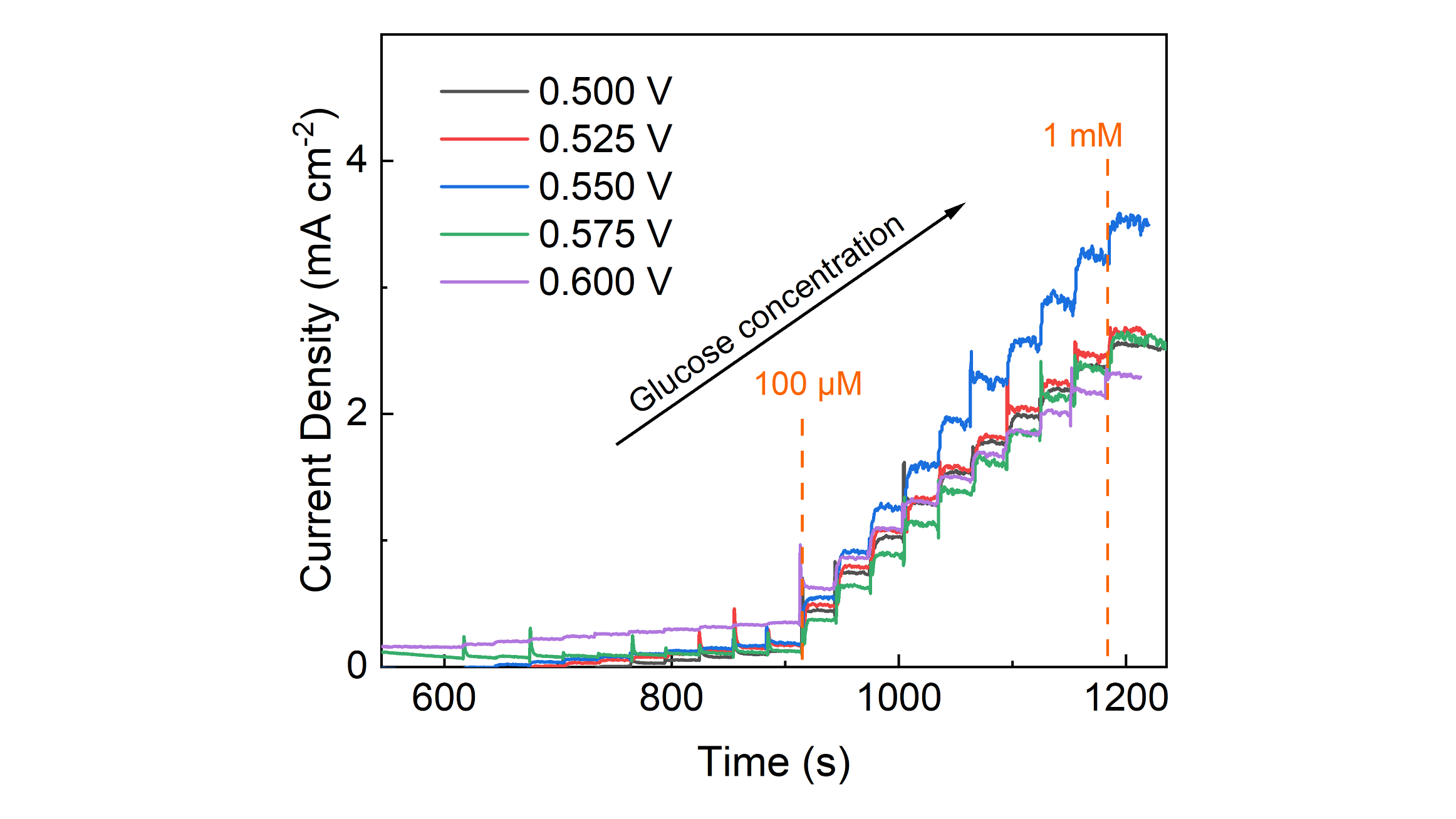


**Fig. S13** Amperometric response of nanoporous Pd-Ni-P MG in the presence of continuously increasing glucose under different potentials.


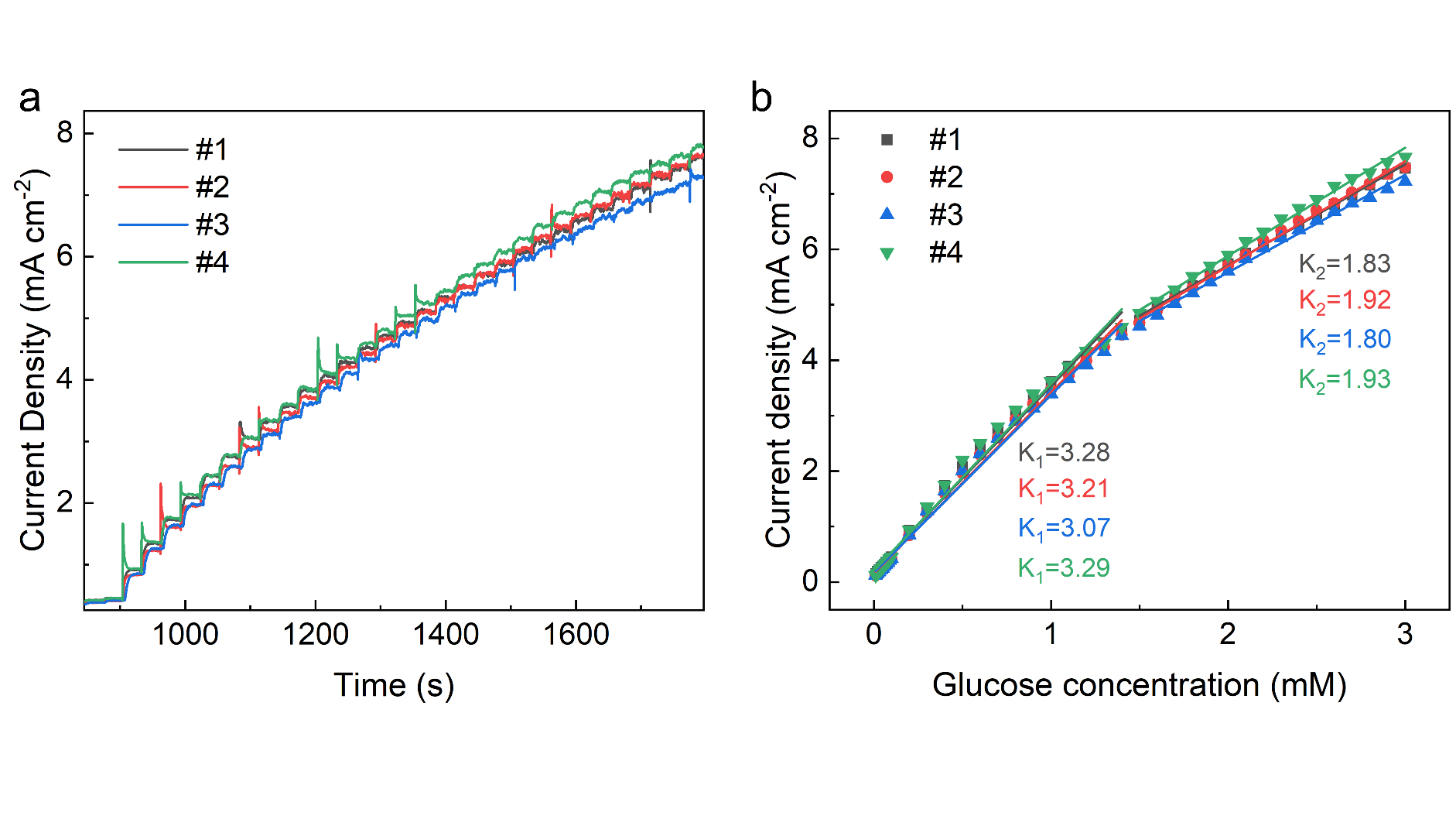


**Fig. S14** (a) The reproducibility experiments of the glucose sensor of the nanoporous Pd-Ni-P MG after 800 seconds of leaching. (b) Linear fitting of current response data from (a).


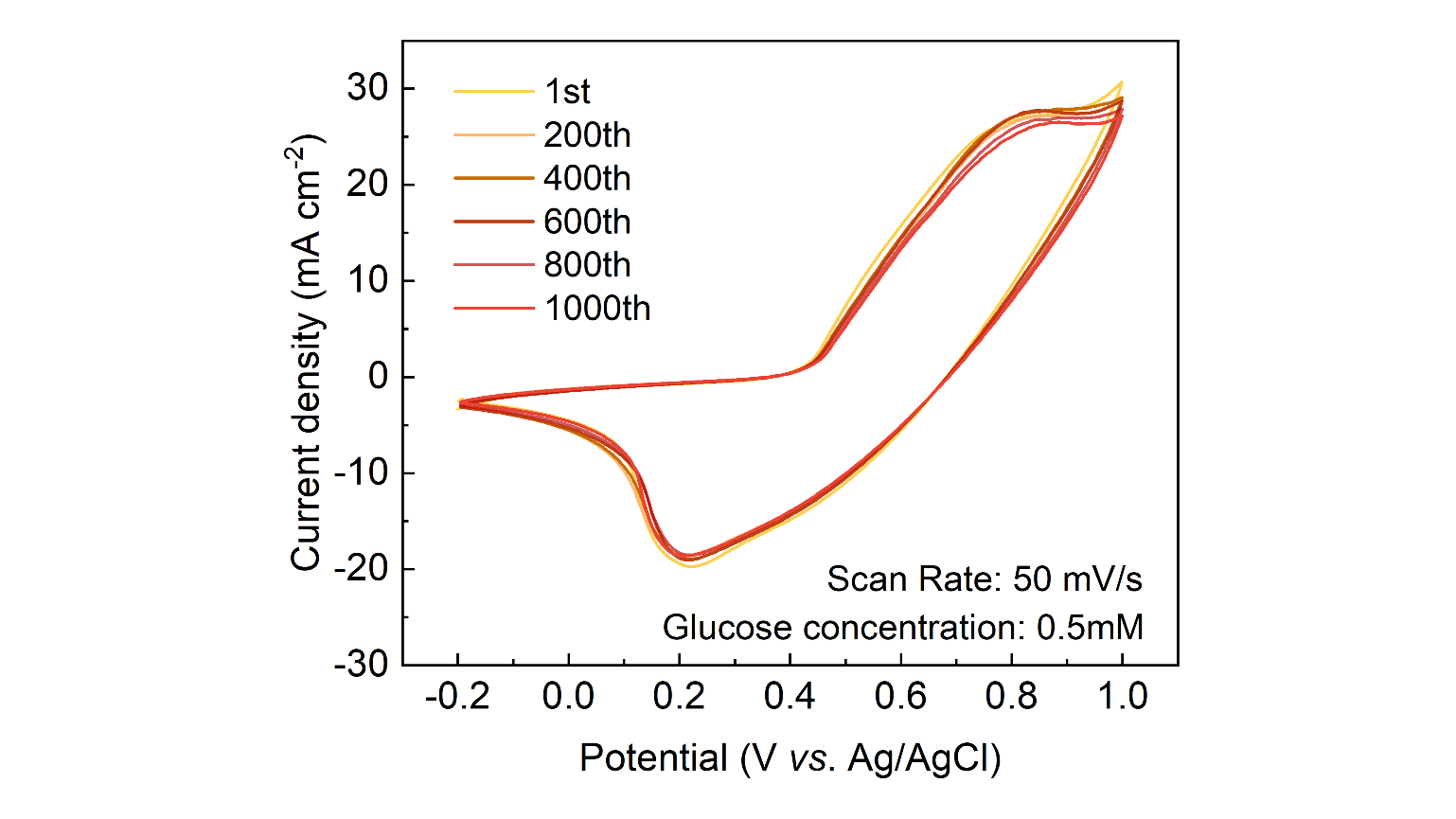


**Fig. S15** The CV curves of the nanoporous Pd-Ni-P MG at different cycles.


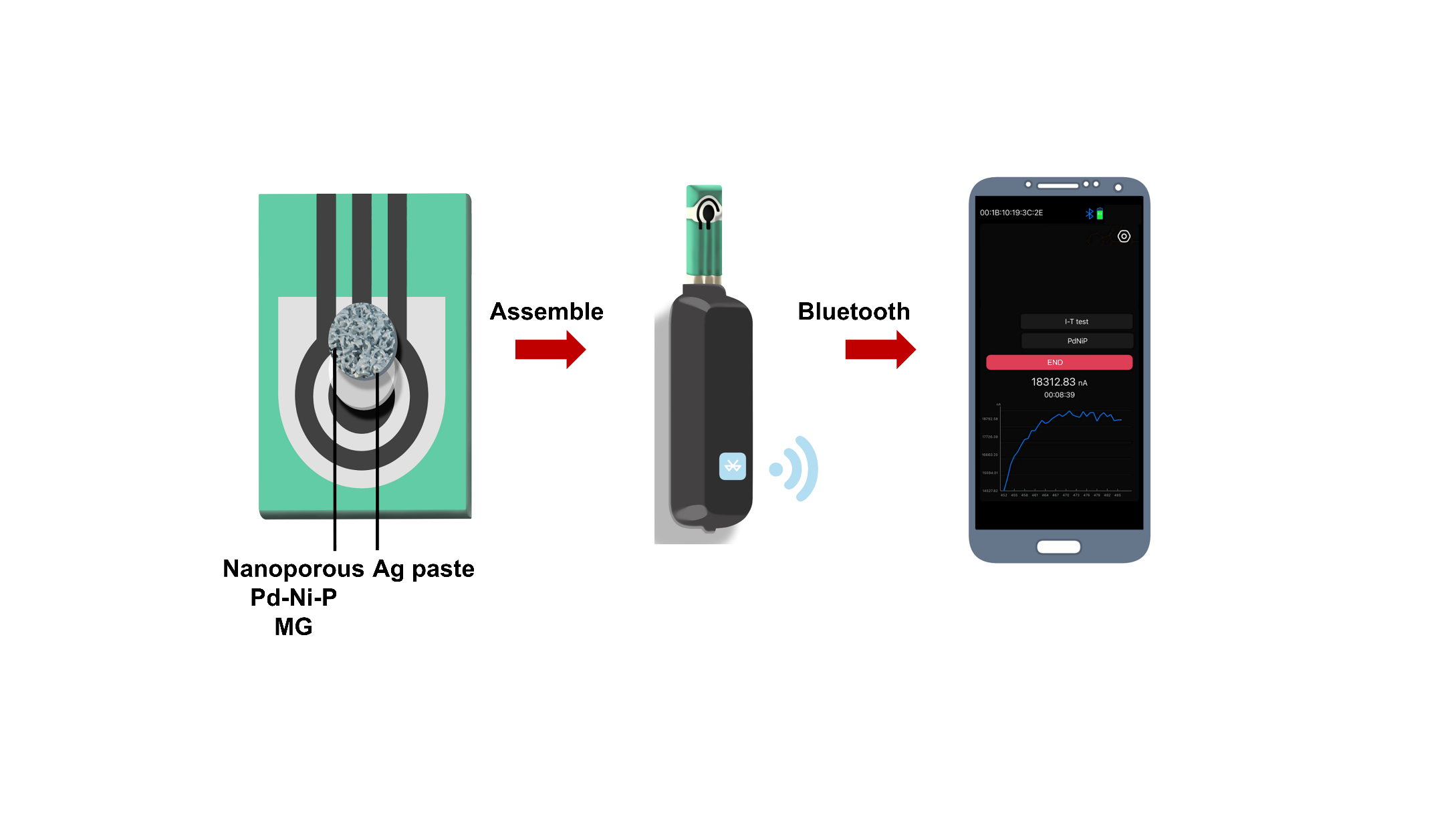


**Fig. S16** The schematic diagram of the nanoporous Pd-Ni-P MG sensor device.


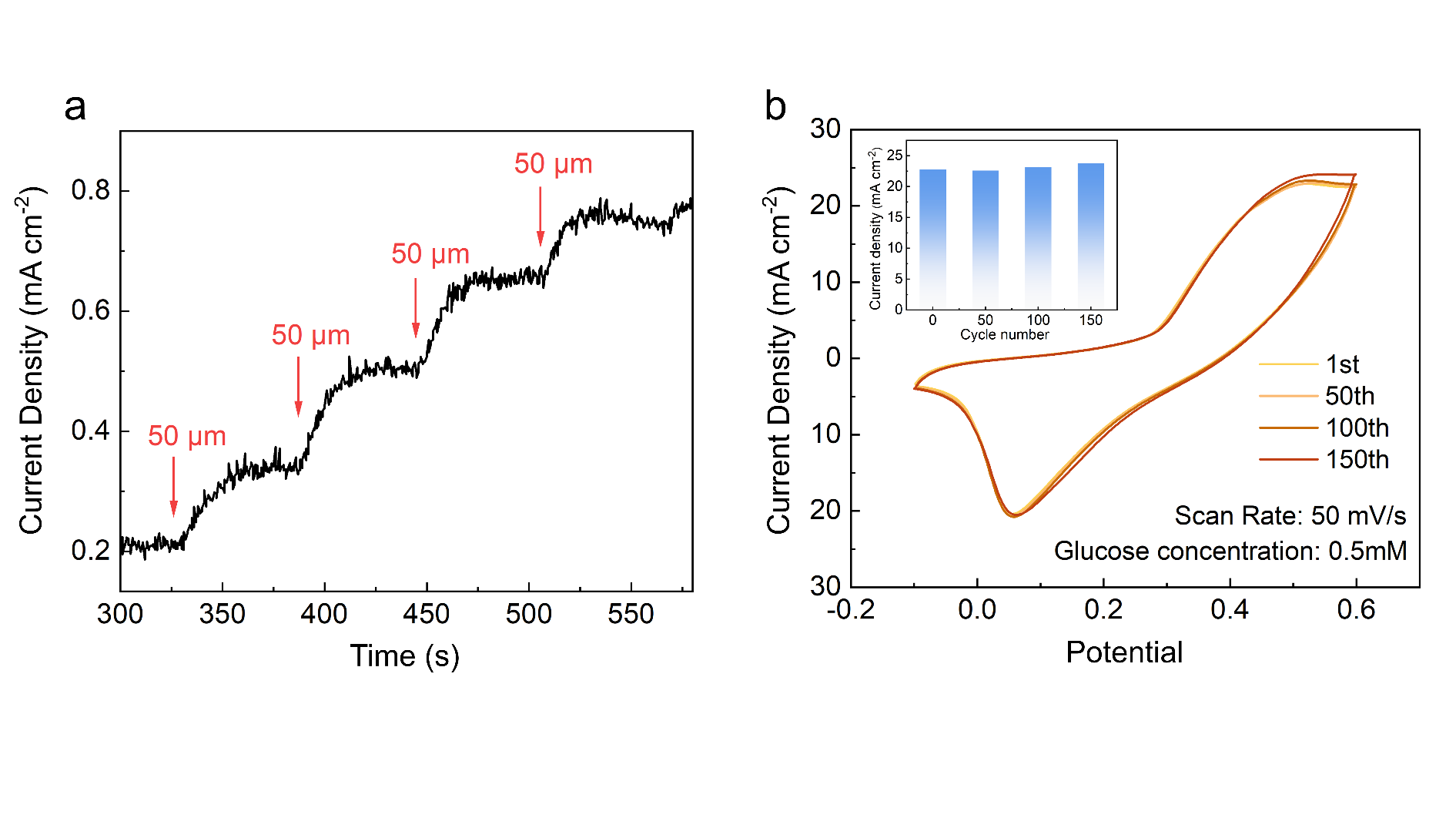


**Fig. S17** (a)The amperometric current response of sensor when exposure to increasing glucose concentration. (b) The CV curves of the nanoporous Pd-Ni-P MG SPE with at different cycles. The insert is the diagram of node current density change.


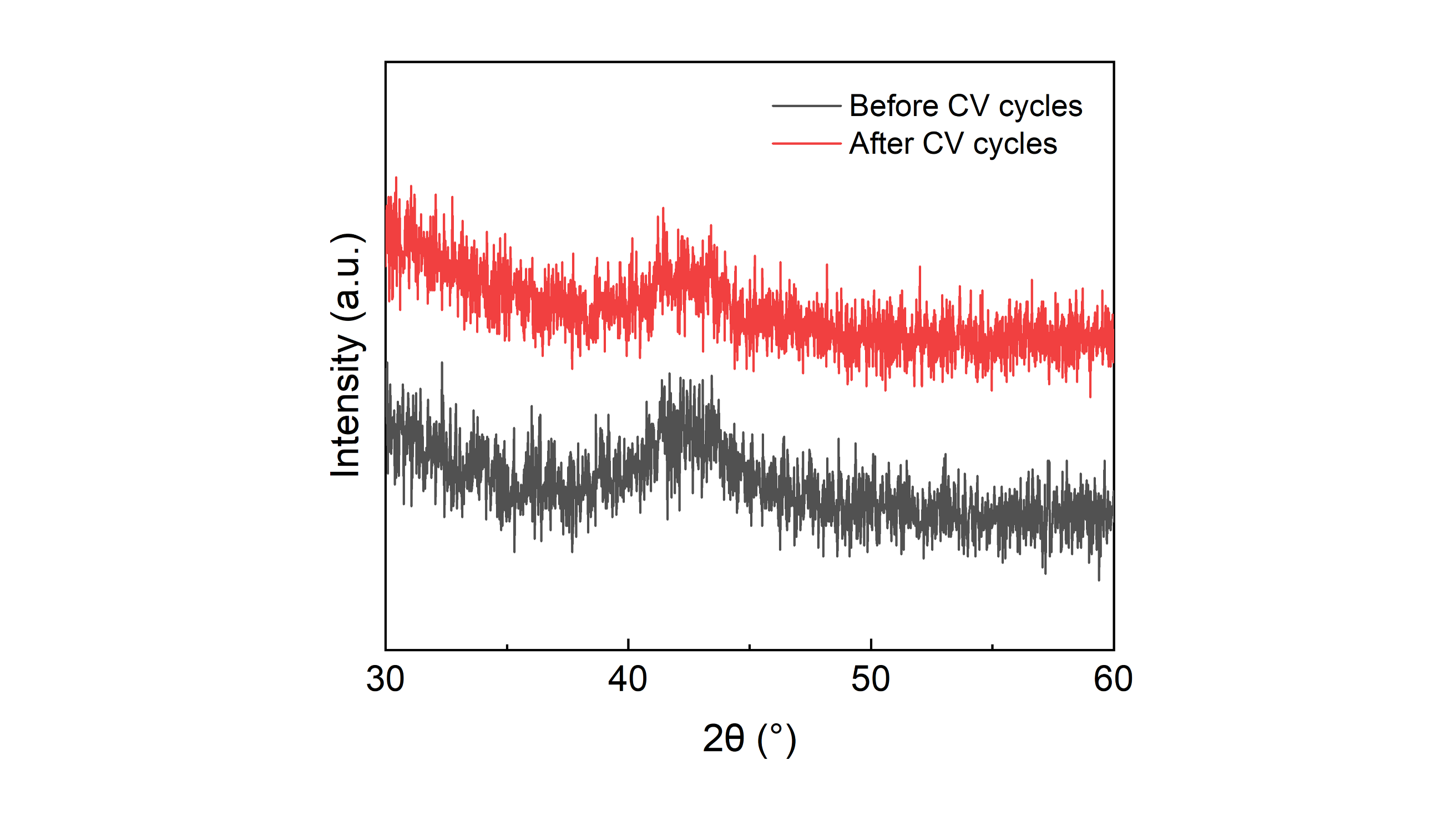


**Fig. S18** The XRD pattern of the nanoporous Pd-Ni-P MG before and after CV cycles.


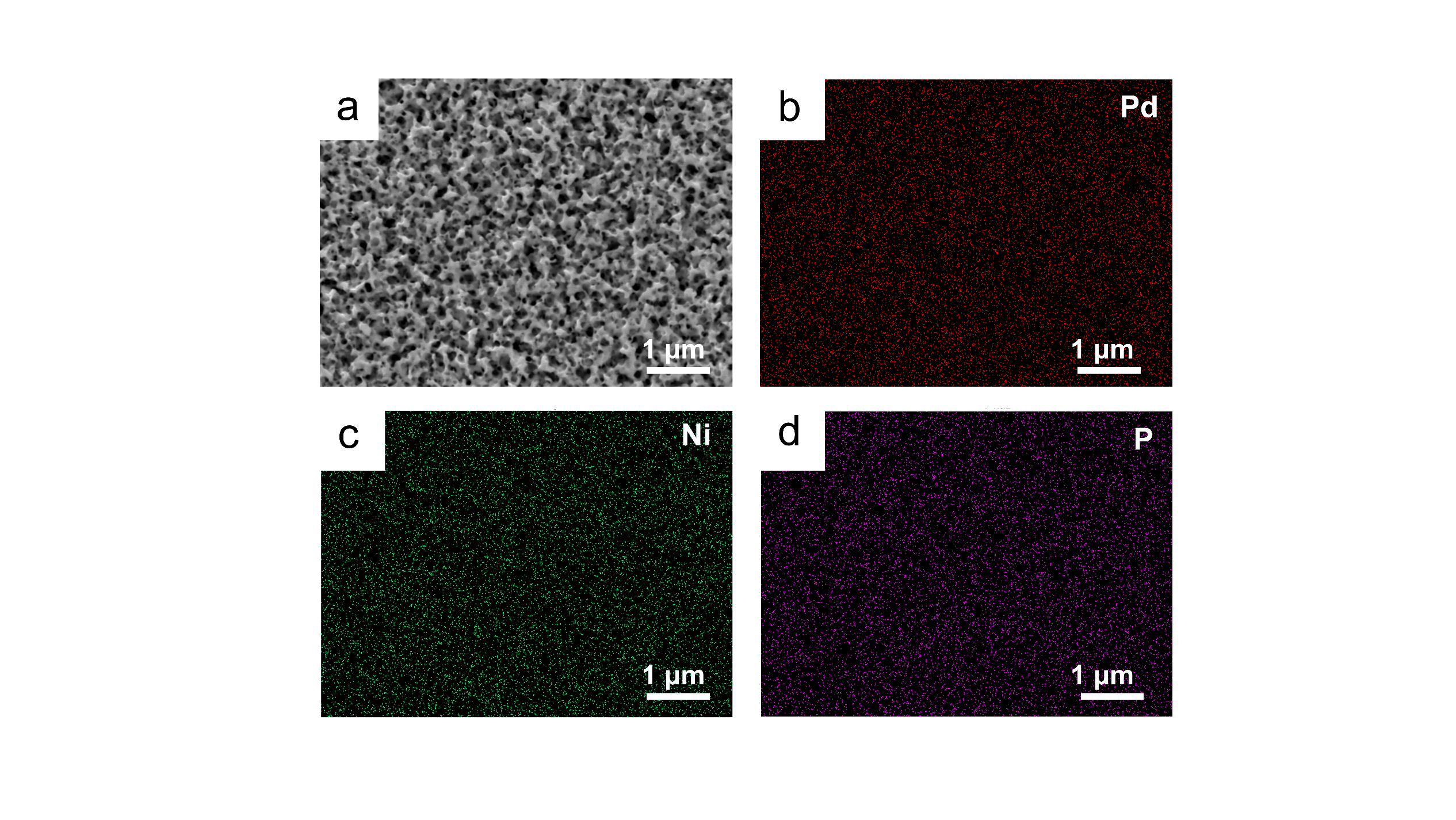


**Fig. S19** (a)The SEM image and (b)- (d) EDS image of nanoporous Pd-Ni-P MG after 1000 CV cycles.

**Table S1.** Atomic percentages of a single cube nanocrystal analyzed by EDS.

| **Ni (at.%)** | **Pd (at.%)** | **P (at.%)** |
| --- | --- | --- |
| 42.9 | 44.1 | 13.0 |

**Table S2.** Atomic percentages of Ni, Pd, and P for nanoporous Pd-Ni-P MG after 200, 400, 800, 1200 seconds of leching analyzed by EDS.

|  | **Ni (at.%)** | **Pd (at.%)** | **P (at.%)** |
| --- | --- | --- | --- |
| Leach 200s | 43.6 | 37.4 | 19.0 |
| Leach 400s | 41.2 | 37.2 | 21.1 |
| Leach 800s | 43.0 | 36.3 | 20.6 |
| Leach 1200s | 41.1 | 37.0 | 21.9 |

**Table S3.** The performance of various non-enzymatic glucose sensors.

| **Glucose sensor** | **Detection limit (μM)** | **Linear range**  **(mM)** | **Sensitivity**  **(μA mM^−1^ cm^−2^)** | **Ref.** |
| --- | --- | --- | --- | --- |
| Cu aerogels | 0.48 | 0.001-1 | 714.3 | ^[10]^ |
| Au_200_Bi | 8.7 | 0.013-3.3 | 664 | ^[11]^ |
| Au@Cu_2_O | 18 | 0.05-2.0 | 715 | ^[12]^ |
| Cu nanowires | 35 | 0.0001-3 | 420.3 | ^[12]^ |
| Pt1/Cu@CuO  NWs | 3.6 | 0.01-5.12 | 852.163 | ^[13]^ |
| Pt/Ni@RGO | 6.3 | 0.02-5 | 171.92 | ^[14]^ |
| Au NFs@CC | 5.18 | 0.005-4 | 63.9 | ^[15]^ |
| CuO nanoparticles | -- | 0.05-5 | 2050 | ^[16]^ |
| ZIF-Zn_0.5_ Co_0.5_ | 9 | up to 1.25 | 1105.6 | ^[17]^ |
| Porous NiO nanostrcuture | 0.084 | 0.005-0.825 | 2632.53 | ^[18]^ |
| CuTF/FTO | 0.025 | 0.001–2 | 2196.6 | ^[19]^ |
| NiCo_2_O_4_ | 2.494  14.356 | 0.001-1  1-6 | 1197  208 | ^[20]^ |
| Pt_1_/Ni(OH)_2_/NG | -- | 0.01-2.18 | 220.75 | ^[21]^ |
| Co-CuS-2 | 0.1 | 0.001–3.66 | 1475.97 | ^[22]^ |
| AgNPs/MoS_2_/PtE | 1000 | 1.0–15.0 | 46.5 | ^[23]^ |
| CuO/Au/TiO_2_ | 4.1 | 0-1  1-10 | 707.1  293.6 | ^[24]^ |
| CNT-CuO NC | 3.90 | 0.005-0.1 | 15.3 | ^[25]^ |
| CuNFs/BDD | 0.2 | 0.2-2 | 2119 | ^[26]^ |
| AuNi@AC nanosensor | 0.41 | 0.05–1.7 | 1955 | ^[27]^ |
| Modified Ni(OH)_2_/Cu  (OH)_2_/SPE | 0.22 | 0.001–2 | 2029 | ^[28]^ |
| Nanoporous Pd-Ni-P MG | 2.94 | 0.001-1.5  1.5-3 | 3190  1910 | This  Work |

**Table S4.** Atomic percentages of Ni, Pd, and P for nanoporous Pd-Ni-P MG after 1000 CV cycles.

|  | **Ni (at.%)** | **Pd (at.%)** | **P (at.%)** |
| --- | --- | --- | --- |
| After CV cycles | 43.9 | 34.1 | 22.0 |

**Table S5**. Fitting parameters of the peak fit for T(r) first peaks of nanoporous Pd-Ni-P MG.

| ***i-j*** | ***R_ij_* (Å)** | ***W_ij_*** |
| --- | --- | --- |
| Pd-Pd | 2.82 | 0.24 |
| Ni-Pd | 2.69 | 0.38 |
| Ni-P | 2.28 | 0.093 |
| Pd-P | 2.41 | 0.119 |
| Ni-Ni | 2.56 | 0.15 |
| P-P | 2.00 | 0.01 |

**Table S6**. The Gaussian fitting results of the second coordination shell of nanoporous Pd-Ni-P MG.

| **Connection mode** | **Before CV cycles** | **After CV cycles** |
| --- | --- | --- |
| 1-atom | 34.6947% | 40.7162% |
| 2-atom | 36.3950% | 32.6403% |
| 3-atom | 25.1300% | 21.5180% |
| 4-atom | 3.7802% | 5.1255% |

1. **Reference**

[1] S. Lan, L. Zhu, Z. Wu, L. Gu, Q. Zhang, H. Kong, J. Liu, R. Song, S. Liu, G. Sha, Y. Wang, Q. Liu, W. Liu, P. Wang, C.-T. Liu, Y. Ren, X.-L. Wang, *Nat. Mater.* **2021**, *20*, 1347.

[2] X. Qiu, J. W. Thompson, S. J. Billinge, *J. Appl. Crystallogr.* **2004**, *37*, 678.

[3] H. Zhu, Y. Huang, J. Ren, B. Zhang, Y. Ke, A. K. ‐Y. Jen, Q. Zhang, X. Wang, Q. Liu, *Adv. Sci.* **2021**, *8*, 2003534.

[4] D. Ma, A. D. Stoica, L. Yang, X.-L. Wang, Z. P. Lu, J. Neuefeind, M. J. Kramer, J. W. Richardson, Th. Proffen, *Applied Physics Letters* **2007**, *90*, 211908.

[5] J. Ding, E. Ma, M. Asta, R. O. Ritchie, *Sci. Rep.* **2015**, *5*, 17429.

[6] K. K. Naik, A. Gangan, B. Chakraborty, S. K. Nayak, C. S. Rout, *ACS Appl. Mater. Interfaces* **2017**, *9*, 23894.

[7] W. Lu, S. Xue, X. Liu, C. Bao, H. Shi, *Microchem. J.* **2024**, *196*, 109606.

[8] S. Lan, Y. Ren, X. Y. Wei, B. Wang, E. P. Gilbert, T. Shibayama, S. Watanabe, M. Ohnuma, X.-L. Wang, *Nat. Commun.* **2017**, *8*, 14679.

[9] S. Lan, Z. D. Wu, M. T. Lau, H. W. Kui, *J. Non-Cryst. Solids* **2013**, *373*, 5.

[10] Q. Fang, H. Wang, X. Wei, Y. Tang, X. Luo, W. Xu, L. Hu, W. Gu, C. Zhu, *Adv. Healthcare Mater.* **2023**, *12*, 2301073.

[11] Q. Fang, Y. Qin, H. Wang, W. Xu, H. Yan, L. Jiao, X. Wei, J. Li, X. Luo, M. Liu, L. Hu, W. Gu, C. Zhu, *Anal. Chem.* **2022**, *94*, 11030.

[12] Y. Su, H. Guo, Z. Wang, Y. Long, W. Li, Y. Tu, *Sens. Actuators, B* **2018**, *255*, 2510.

[13] Y. Zhao, Y. Jiang, Y. Mo, Y. Zhai, J. Liu, A. C. Strzelecki, X. Guo, C. Shan, *Small* **2023**, *19*, 2207240.

[14] R. Ayranci, B. Demirkan, B. Sen, A. Şavk, M. Ak, F. Şen, *Mater. Sci. Eng., C* **2019**, *99*, 951.

[15] Z. Zhao, T. Wang, K. Li, D. Long, J. Zhao, F. Zhu, W. Gong, *Sens. Actuators, B* **2023**, *388*, 133798.

[16] B. Fan, B. D. Spindler, W. Zhao, H. Chan, Z. Wang, M. Kim, Y. Chipangura, P. Bühlmann, A. Stein, *ACS Appl. Nano Mater.* **2023**, *6*, 1475.

[17] K. Kim, J. Kim, Y.-S. Bae, *ACS Sustainable Chem. Eng.* **2022**, *10*, 11702.

[18] P. Chakraborty, N. Deka, D. C. Patra, K. Debnath, S. P. Mondal, *Surf. Interfaces* **2021**, *26*, 101324.

[19] J. Lee, H. Kong, H. Kim, S. Ko, J. Mun, J. Yeo, *ACS Appl. Electron. Mater.* **2024**, *6*, 1274.

[20] L. Wang, X. Lv, L. Zhang, Y. Fang, H. Wang, J. Ren, *Molecules* **2022**, *27*, 7745.

[21] B. Long, Y. Zhao, P. Cao, W. Wei, Y. Mo, J. Liu, C.-J. Sun, X. Guo, C. Shan, M.-H. Zeng, *Anal. Chem.* **2022**, *94*, 1919.

[22] D. Zhang, X. Zhang, Y. Bu, J. Zhang, R. Zhang, *Nanomaterials* **2022**, *12*, 1394.

[23] T. D. Van, N. D. T. Thuy, T. D. V. Phuong, N. N. Thi, T. N. Thi, T. N. Phuong, T. V. Van, H. Vuong-Pham, T. P. Dinh, *Curr. Appl Phys.* **2022**, *43*, 116.

[24] W. Zhu, L. Qiu, Y. Wu, M. Wang, L. Qin, S. Wu, X. Li, *IEEE Sens. J.* **2024**.

[25] M. Geetha, M. R. Maurya, S. Al-maadeed, A. A. Muthalif, K. K. Sadasivuni, *J. Electron. Mater.* **2022**, *51*, 4905.

[26] Q. Gao, W. Zhang, C. Zhao, W. Yan, S. Han, Y. Li, Q. Liu, X. Li, D. Liu, *Adv. Mater. Interfaces* **2022**, *9*, 2200034.

[27] K. Arikan, H. Burhan, E. Sahin, F. Sen, *Chemosphere* **2022**, *291*, 132718.

[28] M. L. Chelaghmia, H. Fisli, M. Nacef, D. A. Brownson, A. M. Affoune, H. Satha, C. E. Banks, *Anal. Methods* **2021**, *13*, 2812.
